# Conditional deletion of caspase-8 in macrophages alters macrophage activation in a RIPK-dependent manner

**DOI:** 10.1186/s13075-015-0794-z

**Published:** 2015-10-16

**Authors:** Carla M. Cuda, Alexander V. Misharin, Sonal Khare, Rana Saber, FuNien Tsai, Amy M. Archer, Philip J. Homan, G. Kenneth Haines, Jack Hutcheson, Andrea Dorfleutner, G. R. Scott Budinger, Christian Stehlik, Harris Perlman

**Affiliations:** Division of Rheumatology, Department of Medicine, Feinberg School of Medicine, Northwestern University, 240 East Huron Street, Room M300, Chicago, IL 60611 USA; Department of Pathology, Icahn School of Medicine at Mount Sinai, New York, NY 10029 USA; Division of Rheumatology, Department of Medicine, University of Texas Southwestern Medical Center, Dallas, TX 75390 USA; Division of Pulmonary and Critical Care Medicine, Department of Medicine, Feinberg School of Medicine, Northwestern University, Chicago, IL 60611 USA

## Abstract

**Introduction:**

Although caspase-8 is a well-established initiator of apoptosis and suppressor of necroptosis, recent evidence suggests that this enzyme maintains functions beyond its role in cell death. As cells of the innate immune system, and in particular macrophages, are now at the forefront of autoimmune disease pathogenesis, we examined the potential involvement of caspase-8 within this population.

**Methods:**

*Cre*^LysM^*Casp8*^fl/fl^ mice were bred via a cross between *Casp8*^fl/fl^ mice and *Cre*^LysM^ mice, and *RIPK3*^−/−^*Cre*^LysM^*Casp8*^fl/fl^ mice were generated to assess the contribution of receptor-interacting serine-threonine kinase (RIPK)3. Immunohistochemical and immunofluorescence analyses were used to examine renal damage. Flow cytometric analysis was employed to characterize splenocyte distribution and activation. *Cre*^LysM^*Casp8*^fl/fl^ mice were treated with either Toll-like receptor (TLR) agonists or oral antibiotics to assess their response to TLR activation or TLR agonist removal. Luminex-based assays and enzyme-linked immunosorbent assays were used to measure cytokine/chemokine and immunoglobulin levels in serum and cytokine levels in cell culture studies. In vitro cell culture was used to assess macrophage response to cell death stimuli, TLR activation, and M1/M2 polarization. Data were compared using the Mann–Whitney *U* test.

**Results:**

Loss of caspase-8 expression in macrophages promotes onset of a mild systemic inflammatory disease, which is preventable by the deletion of RIPK3. In vitro cell culture studies reveal that caspase-8–deficient macrophages are prone to a caspase-independent death in response to death receptor ligation; yet, caspase-8–deficient macrophages are not predisposed to unchecked survival, as analysis of mixed bone marrow chimeric mice demonstrates that caspase-8 deficiency does not confer preferential expansion of myeloid populations. Loss of caspase-8 in macrophages dictates the response to TLR activation, as injection of TLR ligands upregulates expression of costimulatory CD86 on the Ly6C^high^CD11b^+^F4/80^+^ splenic cells, and oral antibiotic treatment to remove microbiota prevents splenomegaly and lymphadenopathy in *Cre*^LysM^*Casp8*^fl/fl^ mice. Further, caspase-8–deficient macrophages are hyperresponsive to TLR activation and exhibit aberrant M1 macrophage polarization due to RIPK activity.

**Conclusions:**

These data demonstrate that caspase-8 functions uniquely in macrophages by controlling the response to TLR activation and macrophage polarization in an RIPK-dependent manner.

**Electronic supplementary material:**

The online version of this article (doi:10.1186/s13075-015-0794-z) contains supplementary material, which is available to authorized users.

## Introduction

Caspase-8 is intimately involved in two essential death pathways—apoptosis and necroptosis—that are responsible for the fate of a cell. Stimulation of a death receptor (DR), such as Fas or tumor necrosis factor (TNF) receptor 1, by its ligand promotes recruitment of Fas-associated death domain protein (FADD) [1]. This protein aggregation facilitates recruitment of the cysteine-aspartic acid enzyme pro-caspase-8, which becomes active upon dimerization. Active caspase-8 initiates the degradative phase of apoptosis through activation of caspases-3/7 or blocks necroptosis via suppression of receptor-interacting serine/threonine protein kinase (RIPK)1-RIPK3 signaling, depending upon the availability of cellular FADD-like interleukin (IL)-1β-converting enzyme-inhibitory protein (cFLIP) [1, 2]. Low levels of cFLIP allow caspase-8 homodimers to form, and apoptosis ensues [2]. Conversely, high levels of cFLIP enable formation of caspase-8-cFLIP heterodimers, which limit RIPK1-RIPK3 signaling for necroptosis and prevent apoptosis [2]. In the absence of caspase-8, apoptosis may not occur, but RIPK1-RIPK3 signaling proceeds unchecked, leading to necroptosis [2]. Further, evidence is emerging that suggests that caspase-8 also maintains cell type–specific, RIPK-dependent, cell death–independent functions, including interferon regulatory factor IRF3 processing for degradation [3, 4], IL-1β production [5, 6], inflammasome activation [5, 7], and Toll-like receptor (TLR) signaling [8].

We previously showed that conditional deletion of Fas or caspase-8 specifically in innate immune cells supports a role for these signaling components in cell activation. An aggressive systemic lupus erythematosus (SLE)-like disease develops in mice following myeloid cell–specific deletion of Fas (*Cre*^LysM^*Fas*^fl/fl^) [9] or dendritic cell (DC)-specific deletion of caspase-8 (*Cre*^CD11c^*Casp8*^fl/fl^) [10]. *Cre*^LysM^*Fas*^fl/fl^ are also more susceptible than control mice to lipopolysaccharide (LPS)-induced shock [9]. In addition, caspase-8–deficient bone marrow–derived dendritic cells (BMDCs) are hyperresponsive to TLR activation in an RIPK1-dependent manner, and *Cre*^CD11c^*Casp8*^fl/fl^ splenic DCs upregulate costimulatory molecules in response to in vivo administration of TLR agonists [10]. Further, deletion of MyD88 in *Cre*^CD11c^*Casp8*^fl/fl^ mice prevents SLE-like disease, although oral antibiotic treatment is ineffective at diminishing inflammatory phenotypes [10]. Because these data reveal a cell-specific role for Fas in myeloid cells and caspase-8 in DCs in the regulation of TLR signaling, we evaluated the consequences of caspase-8 deletion in the myeloid cell compartment.

We now document that caspase-8 functions in myeloid cells to maintain macrophage activation, in part through RIPK1 and RIPK3. Specific deletion of caspase-8 in myeloid cells (*Cre*^LysM^*Casp8*^fl/fl^) leads to the development of a mild systemic inflammation characterized by splenomegaly, lymphadenopathy, immune complex deposition in the kidney, proteinuria, hypergammaglobulinemia, and elevated amounts of serum cytokines that is preventable by RIPK3 deletion. Although Ly6C^high^ and Ly6C^low^ CD11b^+^F4/80^+^ splenic populations are increased in *Cre*^LysM^*Casp8*^fl/fl^ mice, these cells are insufficient inducers of antigen-specific T-cell proliferation. In vitro cell culture studies reveal that caspase-8–deficient macrophages are prone to a caspase-independent death in response to DR ligation; yet, caspase-8–deficient myeloid populations are not predisposed to unchecked survival, as analysis of mixed bone marrow chimeric mice demonstrates that caspase-8 deficiency does not confer preferential expansion of myeloid populations. Despite the relatively mild inflammatory phenotype of *Cre*^LysM^*Casp8*^fl/fl^ mice, caspase-8–deficient bone marrow–derived macrophages (BMDMs) are hyperresponsive to TLR activation in an RIPK1-dependent manner. Further, myeloid cell–specific caspase-8 deficiency appears to dictate the in vivo response to TLR activation, as injection of TLR ligands into *Cre*^LysM^*Casp8*^fl/fl^ mice upregulates the expression of costimulatory CD86 on the Ly6C^high^CD11b^+^F4/80^+^ splenic population. In addition, oral antibiotic treatment prevents inflammatory disease phenotypes in young *Cre*^LysM^*Casp8*^fl/fl^ mice. Moreover, caspase-8 controls the polarization of macrophages in response to M1-skewing media in an RIPK1-dependent manner. These data document a role for caspase-8 as a regulator of both the TLR response and macrophage polarization via limiting RIPK in macrophages.

## Methods

### Mice

C57BL/6 (B6) mice homozygous for *loxP*-flanked caspase-8 allele (*Casp8*^fl/fl^) [11] were crossed with mice expressing Cre under control of murine lysozyme M gene promoter (*Cre*^LysM^; The Jackson Laboratory, Bar Harbor, ME, USA), generating *Cre*^LysM^*Casp8*^fl/fl^ mice. *Cre*^LysM^*Casp8*^fl/fl^ mice were crossed with *RIPK3*^−/−^ (Genentech, South San Francisco, CA, USA) to generate *RIPK3*^−/−^*Cre*^LysM^*Casp8*^fl/fl^ mice. *OT-II/RAG*^−/−^ and B6.*CD45.1* were purchased from The Jackson Laboratory. B6.*CD45.1/2* mice were generated from a cross of B6 (The Jackson Laboratory) and B6.*CD45.*1 mice. Female mice were used in all studies. Proteinuria was assessed using Uristix reagent strips (Siemens Healthcare Diagnostics, Tarrytown, NY, USA). Transnetyx (Cordova, TN, USA) performed all genotyping of mice. All animal experiments were approved by the Northwestern University Institutional Animal Care and Use Committee.

### Histopathologic studies

Paraffin-embedded kidney sections (5 μm) were treated with periodic acid–Schiff stain, and a pathologist blinded to the study scored kidney sections using an Olympus BS40 microscope (Olympus Life Science, Center Valley, PA, USA) as previously described [12]. Frozen kidney sections (10 μm) were stained with anti-IgG-fluorescein isothiocyanate [12]. All images were photographed at × 40, ×200, ×400, or × 600 magnification on an Olympus BX41 microscope equipped with an Olympus DP20 camera.

### Flow cytometry

Surface staining of cell suspensions and gating strategies were carried out as previously described [9, 10, 13]. At least 100,000 events were captured on a BD LSR II flow cytometer (BD Biosciences, San Jose, CA, USA). Data were analyzed with FlowJo software (Tree Star, Ashland, OR, USA). Dead cells were excluded using the Molecular Probes LIVE/DEAD Fixable Aqua Dead Cell Stain Kit (Life Technologies, Carlsbad, CA, USA). For cell-sorting studies, splenocytes preincubated with Fc block antibody were stained with fluorescent antibodies (information available upon request). Splenocyte populations sorted on a BD FACSAria II instrument (BD Biosciences) at the University of Chicago and Northwestern University Cancer Center Flow Core had an average purity of 97 %.

### Mixed bone marrow chimeras

Bone marrow was aseptically harvested from tibias, femurs, and humeri from 9-week-old mice and erythrocytes were lysed (BD Pharm Lyse buffer; BD Biosciences). Cells were incubated with Fc block antibody followed by incubation with fluorochrome-conjugated antibodies against B220, CD4, CD8, CD11b, Ly6G, NK1.1, Siglec F, Ter119, c-Kit, and Sca-1 (BD Biosciences; and eBioscience and BioLegend, both located in San Diego, CA, USA). Cell suspensions were subjected to fluorescence-activated cell sorting (FACS) analysis to obtain the Lin^−^Sca-1^+^c-kit^+^ (LSK) cell population. Three-month-old B6.*CD45.1/2* received a single 1000-cGy γ-irradiation dose using a Cs-137-based Gammacell 40 irradiator (Best Theratronics, Ottawa, ON, Canada). After 12 h, 5 × 10^5^ LSK cells were intravenously injected from *Casp8*^fl/fl^, *Cre*^LysM^*Casp8*^fl/fl^, a mixture of *Casp8*^fl/fl^ plus B6.*CD45.1* or *Cre*^LysM^*Casp8*^fl/fl^ plus B6.*CD45.1* (1:1 ratio). Chimeric mice were maintained on trimethoprim/sulfamethoxazole (40 mg/5 mg, respectively; Hi-Tech Pharmacal/Akorn, Amityville, NY, USA) diluted in autoclaved water (2 ml antibiotics/500 ml water) and phenotyped 8 months posttransfer.

### In vitro assays

For mixed leukocyte reactions, splenocytes were incubated with anti-CD19 beads and negative fractions were incubated with anti-CD11b magnetic-activated cell sorting beads (Miltenyi Biotec, Bergisch Gladbach, Germany) to purify antigen-presenting cells (APCs). Purified APCs were pulsed with 10 μg/ml ovalbumin (OVA) peptide (amino acids 323–339) for 60 minutes at 37 °C. OVA-specific splenic CD4^+^ T cells were isolated from B6.*CD45.1/OT-II/RAG*^−/−^ mice using CD4^+^ T-cell isolation kits (Miltenyi Biotec) according to the manufacturer’s instructions. Purity of APCs and T cells was 90 %. T cells were labeled with carboxyfluorescein diacetate succinimidyl ester (CFSE) (500 nM for 12 minutes at 37 °C; Invitrogen, Carlsbad, CA, USA). Pulsed APCs at various ratios were incubated with 2 × 10^5^ CFSE-labeled T cells with or without 5 μg/ml class B CpG (ODN 1668; InvivoGen, San Diego, CA, USA) in triplicate in 96-well flat-bottomed plates at 37 °C for 3 days. Cell clusters were dissociated with 7.5 mM ethylenediaminetetraacetic acid for 15 minutes, and stained with anti-CD4 (BD Biosciences). 7-Aminoactinomycin D (0.25 mg/test; BD Biosciences) was used to exclude dead cells. A constant number of CaliBRITE beads (BD Biosciences) were added for acquisition of equal parts in each culture. Live T cells were gated, and the number of divided cells showing less than maximal CFSE fluorescence intensity was determined.

BMDMs were generated by culturing bone marrow in media (Dulbecco’s modified Eagle’s medium with 10 % fetal calf serum, 2 mM glutamine, 100 U penicillin/0.1 mg streptomycin/1 ml and 1 mM sodium pyruvate) plus macrophage colony-stimulating factor (M-CSF; 100 ng/ml). On day 6, cells were replated at 1.75 × 10^6^ cells/well in a 6-well plate, and on day 7 cells were stimulated with the indicated treatments. For cytokine levels, BMDMs were stimulated for 3, 6, and 12 h with LPS (10 ng/ml), imiquimod (5 μg/ml), or CpG (5 μg/ml) with or without necrostatin-1 (Nec-1; 30 μM), and cells were harvested for cytokine transcript levels while supernatants were evaluated for cytokine levels (see below). Following TLR stimulation for the indicated time, ATP (5 mM; Sigma-Aldrich, St. Louis, MO, USA) was added to BMDMs for 45 minutes for evaluation of IL-1β levels in supernatants. For BMDM death assays, BMDMs at a concentration of 2 × 10^6^ cells/ml were stimulated for 48 h with superFasL (100 ng/ml; Enzo Life Sciences, Farmingdale, NY, USA), etoposide (10 μM; Alexis Biochemicals, Lausen, Switzerland) with or without carbobenzoxy-valyl-alanyl-aspartyl-[*O*-methyl]-fluoromethylketone (Z-VAD-FMK, 20 μM; Promega, Madison, WI, USA), and supernatants were evaluated for lactate dehydrogenase (LDH) activity according to the manufacturer’s instructions (Sigma-Aldrich). For splenocyte death assays, 3 × 10^6^ total splenocytes were treated for 10 h with superFasL (100 ng/ml; Enzo Life Sciences) or etoposide (10 μM; Alexis Biochemicals) and stained with annexin-V and Molecular Probes LIVE/DEAD Fixable Aqua Dead Cell Stain. For macrophage polarization assays, BMDMs were cultured with M1-polarizing conditions (primed overnight with interferon [IFN]-γ 100 ng/ml and stimulated for 3 h with LPS [10 ng/ml]) and M2-polarizing conditions (stimulated for 24 h with IL-4 [40 ng/mL]) with or without Nec-1 (30 μM) and/or Z-Ile-Glu(O-ME)-Thr-Asp(O-Me)-FMK (Z-IETD-FMK, 20 μM; BD Biosciences). Polarized BMDMs were lysed directly in QuantiGene Lysis Mixture, and gene expression profiles were determined using a custom QuantiGene 2.0 assay (Affymetrix, Santa Clara, CA, USA) and a Luminex 200 instrument (Luminex Corporation, Austin, TX, USA) (see Additional file 1: Table S1 for panel description).

### In vivo assays

For TLR ligand injection studies, 3-month-old mice were intraperitoneally injected with LPS, imiquimod, or CpG (200 μg/20 g body weight; InvivoGen) and analyzed after 4 h by FACS analysis. For oral antibiotic treatment, 3-week-old mice were given autoclaved water plus ampicillin (1 g/L), vancomycin (0.5 g/L), neomycin sulfate (1 g/L), metronidazole (1 g/L), and sucrose (10 g/L) twice weekly for 8 weeks with no observable weight loss.

### Western blot analysis, antibody/cytokine measurements, and gene expression quantification

Sorted splenocytes and BMDMs were lysed in sample buffer, and equal amounts of proteins were separated by SDS-PAGE, transferred onto polyvinylidene difluoride membranes, and analyzed by immunoblotting with the appropriate primary antibodies (caspase-8, Enzo Life Sciences; glyceraldehyde 3-phosphate dehydrogenase [GAPDH], US Biological, Salem, MA, USA) and horseradish peroxidase (HRP)-conjugated secondary antibodies (goat anti-rat immunogloblulin G [IgG]-HRP; Santa Cruz Biotechnology, Santa Cruz, CA, USA), electrochemiluminescence detection (Pierce/Thermo Scientific, Waltham, MA, USA), and image acquisition (Ultra-Lum, Claremont, CA, USA). Single-stranded DNA (ssDNA)-, double-stranded DNA (dsDNA)-, histone-, or chromatin-reactive IgG antibodies were measured as previously described [12]. Total IgM and IgG isotypes and cytokine/chemokine expression were quantified using Luminex bead-based assays (Affymetrix) and enzyme-linked immunosorbent assays (ELISAs). Total RNA was isolated from BMDMs using the RNeasy Plus Mini Kit according to the manufacturer’s instructions (Qiagen, Valencia, CA, USA). RNA was reverse-transcribed with the High Capacity RNA-to-cDNA kit (Applied Biosystems, Foster City, CA, USA). Real-time PCR was performed on an ABI 7300 real-time PCR machine (Applied Biosystems) using the TaqMan gene expression system (Applied Biosystems) and predesigned fluorescein-labeled primer/probes (β-actin, IL-6: Mm00446190_m1; IL-10: Mm00439614_m1; TNF-α: Mm00443258_m1; IL-1β: Mm00434228_m1; IL-12b: Mm00434174_m1; Life Technologies). The results are presented as relative expression compared with β-actin.

### Data analysis

For analysis of macrophage polarization, data were normalized to expression of housekeeping genes and imported into Partek Genomics Suite V6.6 software (Partek, St. Louis, MO, USA). Differentially expressed genes between the different groups of stimulated macrophages, as well as transcripts with variable expression within the data set, were calculated using one-way analysis of variance (ANOVA). Differentially expressed genes between two analyzed macrophage populations were defined by a Bonferroni-corrected *p* value <0.05 unless stated otherwise. Principal component analysis (PCA) using all transcripts was performed for visualization of sample relationships. Hierarchical clustering of the differentially expressed genes was performed based on a Euclidean algorithm for dissimilarity and average linkage method to determine distance between clusters. All other data are shown as mean ± SD and were compared by Mann–Whitney *U* test using GraphPad Prism 5.0 software (GraphPad Software, San Diego, CA, USA).

## Results

### Mice with conditional deletion of caspase-8 in myeloid cells develop a mild systemic inflammatory disease

**Fig. 1:**
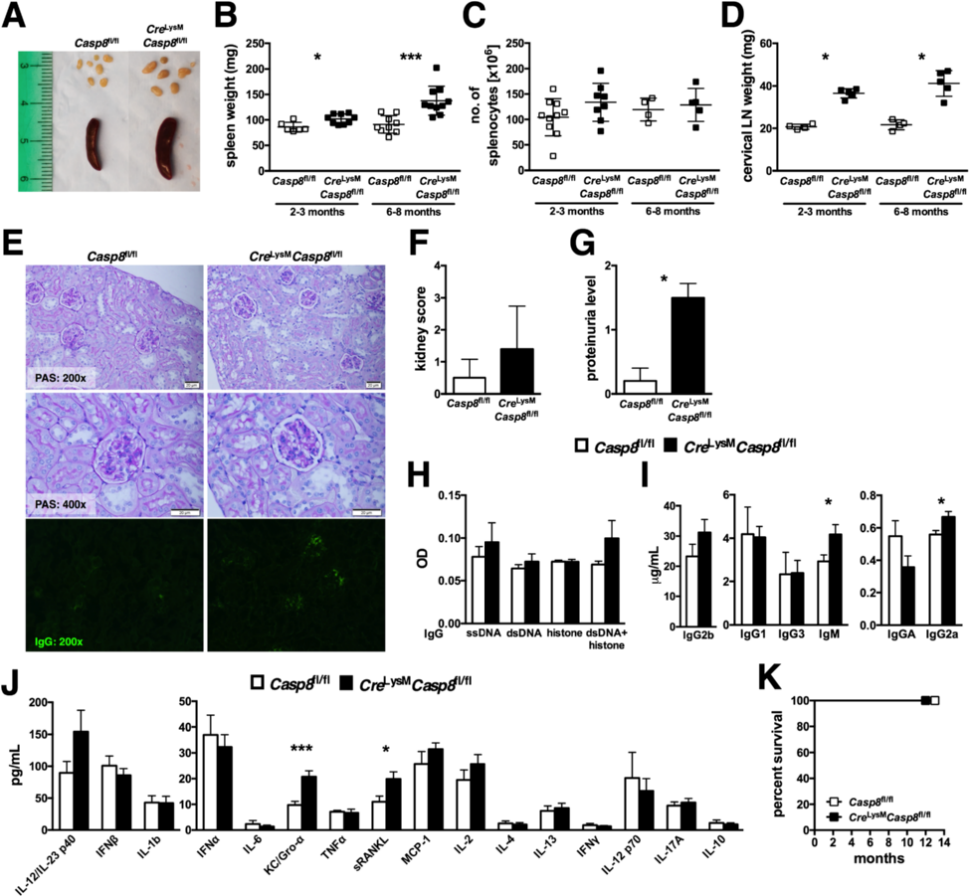
Mice with conditionally deleted caspase-8 exhibit mild systemic inflammation. We evaluated 2–3-month old (young) and 6–8-month-old (aged) female *Casp8* ^fl/fl^ (control) and *Cre* ^LysM^ *Casp8* ^fl/fl^ mice (n ≥ 4) for systemic autoimmune disease phenotypes. **a** Representative spleens and cervical lymph nodes from aged mice. **b** Spleen weights of young and aged mice. **c** Total numbers of live splenocytes from young and aged mice. **d** Cervical lymph node weights of young and aged mice. **e** Formalin-fixed kidney sections (5 μm) of aged mice stained with periodic acid–Schiff (PAS) and frozen kidney sections (10 μm) stained with IgGα-fluorescein isothiocyanate. **f** Kidney score of aged mice. **g** Proteinuria of aged mice assessed using Uristix reagent strips. Serum from aged mice was evaluated for levels of **h** ssDNA-, dsDNA-, histone-, and chromatin-reactive IgG antibodies; **i** total IgM and IgG isotypes; and **j** cytokines and chemokines. **k** Survival study. Data are represented as mean ± SD and were compared by Mann–Whitney *U* test: *p < 0.05; ***p < 0.0005. *Casp8* caspase-8, *dsDNA* double-stranded DNA, *Gro-α* growth-regulated oncogene-α, *IFN* interferon, *Ig* immunoglobulin, *IL* interleukin, *KC* keratinocyte chemoattractant, *LN* lymph node, *MCP* monocyte chemoattractant protein, *OD* optical density, *sRANKL* soluble receptor activator of nuclear factor κB ligand, *ssDNA* single-stranded DNA, *TNF* tumor necrosis factor

The authors of a previous report demonstrated that *Cre*^LysM^*Casp8*^fl/−^ mice failed to generate caspase-8 deficiency in myeloid cells or an observable phenotype [14]. However, in the present study, *Cre*^LysM^*Casp8*^fl/fl^ mice exhibited caspase-8 deletion specifically in myeloid populations. PCR and immunoblot analysis of FACS-sorted splenic neutrophils (CD11b^+^Ly6G^+^) and Ly6C^low^ and Ly6C^high^ CD11b^+^F4/80^+^ cells (Additional file 1: Figure S1a, b) and M-CSF-generated BMDMs (Additional file 1: Figure S2,a, b) from *Cre*^LysM^*Casp8*^fl/fl^ mice showed caspase-8 deletion, whereas lymphocytes and DC populations retained caspase-8. Further, there was no defect in the generation of BMDMs from mice with myeloid cell–specific caspase-8 deficiency (Additional file 1: Figure S2,c, d). Loss of caspase-8 in myeloid cells led to splenomegaly in both young and aged mice (Fig. 1a, b). However, there was no increase in total splenocyte numbers (Fig. 1c). In addition, lymphadenopathy was present in young and aged *Cre*^LysM^*Casp8*^fl/fl^ mice (Fig. 1d). *Cre*^LysM^*Casp8*^fl/fl^ mice failed to develop glomerulonephritis (Fig. 1e, f), although mild IgG deposition in the kidney (Fig. 1e) and higher proteinuria levels (Fig. 1g) were detected compared with control mice. Further, *Cre*^LysM^*Casp8*^fl/fl^ mice displayed elevated levels of IgG2b and total IgM antibodies (Fig. 1i) but did not show evidence of circulating autoreactive antibodies (Fig. 1h). Moreover, serum levels of proinflammatory molecules soluble receptor activator of nuclear factor κB ligand (sRANKL) and keratinocyte chemoattractant/growth-regulated oncogene-α (KC/Gro-α) (Fig. 1j) were elevated compared with levels in control mice. Mortality was similar between *Cre*^LysM^*Casp8*^fl/fl^ and control mice (Fig. 1k). These data indicate that myeloid cell–specific deletion of caspase-8 induces a mild systemic inflammatory disease that may be mediated by enhanced circulating cytokines and chemokines.

### Myeloid cell-specific loss of caspase-8 increases splenic CD11b^+^F4/80^+^ populations

**Fig. 2:**
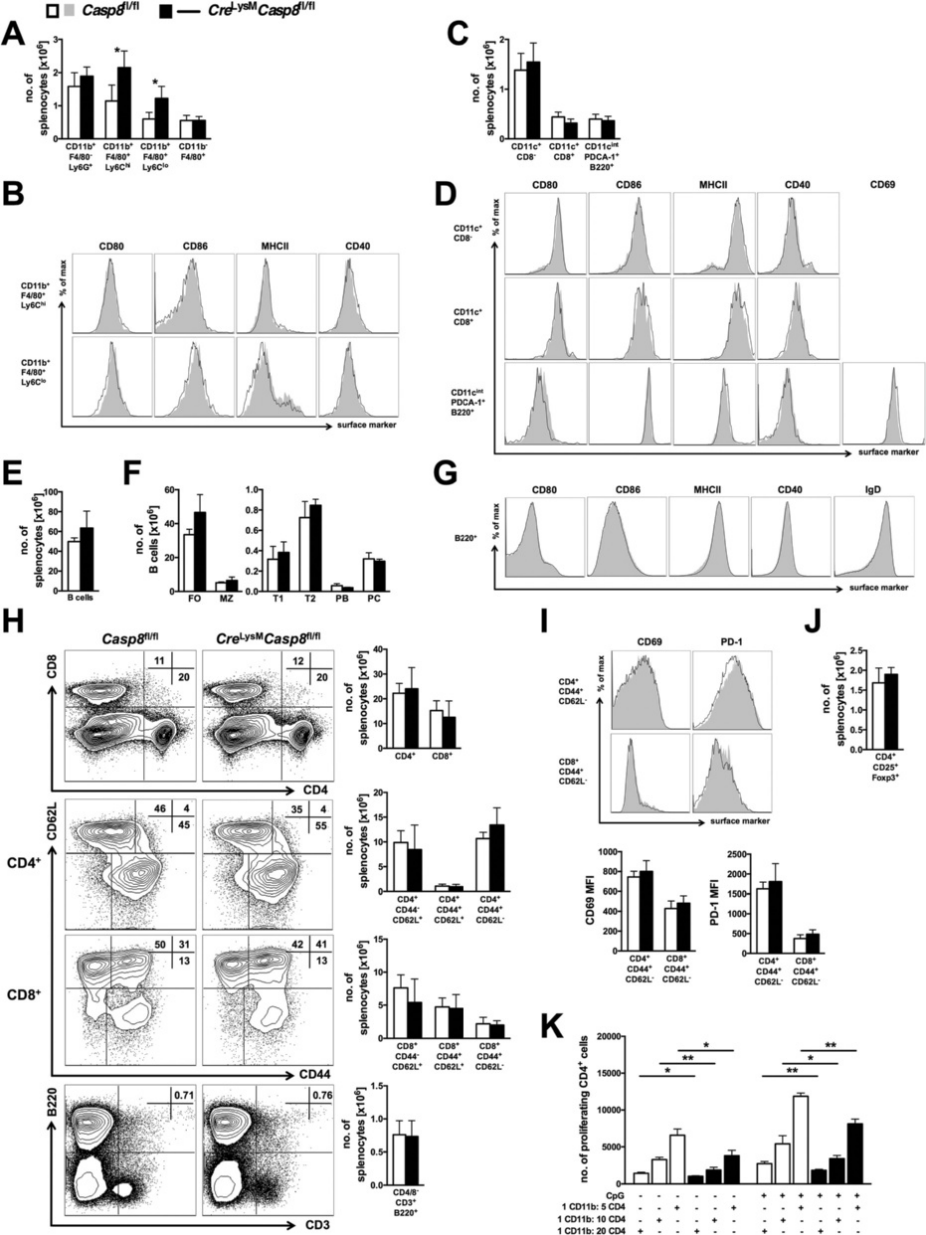
Splenic cellularity and activation profiles of *Cre* ^LysM^ *Casp8* ^fl/fl^ mice are not drastically altered. Splenocytes from 6–8-month-old (aged) female *Casp8* ^fl/fl^ (control) and *Cre* ^LysM^ *Casp8* ^fl/fl^ mice (n ≥ 4) were analyzed by flow cytometry. **a** Number of CD11b^+^F4/80^−^Ly6G^+^ neutrophils, Ly6C^high^ and Ly6C^low^ CD11b^+^F4/80^+^ cells, and CD11b^−^F4/80^+^ red pulp macrophages. **b** Representative fluorescence-activated cell sorting (FACS) plots of CD11b^+^F4/80^+^Ly6C^high^ and CD11b^+^F4/80^+^Ly6C^low^ splenocytes displaying levels of surface activation marker expression. **c** Number of CD11c^+^CD8^−^ and CD11c^+^CD8^−^ conventional DCs and CD11c^intermediate^PDCA-1^+^B220^+^ plasmacytoid DCs. **d** Representative FACS plots of CD11c^+^CD8^−^ and CD11c^+^CD8^−^ conventional DCs and CD11c^intermediate^PDCA-1^+^B220^+^ plasmacytoid DCs displaying levels of surface activation marker expression. **e** Total B-cell (CD11c^−^B220^+^) numbers. **f** B-cell subsets: follicular (FO; CD19^+^CD21/35^+^CD23^+^), marginal zone (MZ; CD19^+^CD21/35^+^CD23^low^), transitional 1 (T1; B220^+^AA4.1^+^CD23^−^), transitional 2 (T2; B220^+^AA4.1^+^CD23^+^), plasmablasts (PB; CD19^+^B220^low^CD138^+^CD21/35^−^CD23^−^), and plasma cells (PC; CD19^+^B220^+^CD138^+^CD21/35^−^CD23^−^). **g** Representative FACS plots from B cells displaying levels of surface activation and maturation marker expression. **h** Representative FACS plots and quantitative graphs of total CD4^+^ and CD8^+^ T-cell numbers, naïve (CD44^−^CD62L^+^), central memory (CD44^+^CD62L^+^) and activated (CD44^+^CD62L^−^) CD4^+^ and CD8^+^ T-cell numbers, and CD4^−^CD8^−^CD3^+^B220^+^ double-negative T-cell numbers. **i** Representative FACS plots and quantitative graphs depicting activated CD4^+^ and CD8^+^ T cells displaying levels of surface activation marker expression. **j** CD4^+^CD25^+^Foxp3^+^ regulatory T-cell numbers. Data are represented as mean ± SD and were compared by Mann–Whitney *U* test: **p < 0.005, ***p < 0.0005. **k** Bead-separated CD11b^+^ cells incubated with ovalbumin were cocultured with B6.*CD45.1/OT-II/RAG* ^−/−^ CD4^+^ T cells at various ratios with or without CpG. Data are represented as mean ± SD of biological triplicates, and experiments were repeated twice. Data were compared by Mann–Whitney *U* test: *p < 0.05; **p < 0.005. *PDCA-1* plasmacytoid dendritic cell antigen 1

To determine the cellular mechanism responsible for the mild inflammatory phenotype that arises in *Cre*^LysM^*Casp8*^fl/fl^ mice, multiparameter flow cytometry was used. *Cre*^LysM^*Casp8*^fl/fl^ mice exhibited increased numbers of Ly6C^high^ and Ly6C^low^ splenic CD11b^+^F4/80^+^ cells, whereas the CD11b^+^F4/80^−^Ly6G^+^ neutrophils and CD11b^−^F4/80^+^ red pulp macrophage populations were not statistically altered compared with control mice (Fig. 2a). Although increased in numbers, no significant alterations in surface expression of activation markers were observed on caspase-8–deficient Ly6C^high^ and Ly6C^low^ splenic CD11b^+^F4/80^+^ cells compared with control populations (Fig. 2b). *Cre*^LysM^*Casp8*^fl/fl^ mice also showed no statistical difference in conventional and plasmacytoid DC numbers (Fig. 2c) or activation status (Fig. 2d) compared with control mice. The lymphocytic populations were then evaluated to determine whether the increased presence of *Cre*^LysM^*Casp8*^fl/fl^ Ly6C^high^ and Ly6C^low^ splenic CD11b^+^F4/80^+^ cells affected B- and T-cell functionality. Loss of caspase-8 in myeloid cells had no observable effect on total B-cell numbers (Fig. 2e), subset distribution (Fig. 2f), or activation and maturation (Fig. 2g). In addition, *Cre*^LysM^*Casp8*^fl/fl^ mice did not exhibit any alterations in CD4^+^ or CD8^+^ total, naïve (CD44^−^CD62L^+^), or activated (CD44^+^CD62L^−^) T cells or in the numbers of double-negative T cells (CD4^−^CD8^−^CD3^+^B220^+^), which are associated with deficiencies in Fas [15–17] (Fig. 2h). Activated CD4^+^ and CD8^+^ T cells expressed similar levels of CD69 and PD-1 (Fig. 2i), and regulatory T-cell numbers were unchanged (Fig. 2j), between *Cre*^LysM^*Casp8*^fl/fl^ and control mice. Additionally, the capacity for antigen presentation to T cells was assessed. Caspase-8–deficient CD11b^+^ cells incubated with OVA peptide suppressed OT-II-specific (C57BL/6-Tg(TcraTcrb)425Cbn/Crl) CD4^+^ T-cell proliferation in the presence or absence of TLR9 activation compared with control CD11b^+^ cells (Fig. 2k). Taken together, these results suggest that, although there are increased numbers of Ly6C^high^ and Ly6C^low^ splenic CD11b^+^F4/80^+^ cells in *Cre*^LysM^*Casp8*^fl/fl^ mice, these cells do not promote heightened activation and functionality of surrounding cells and in fact are capable of suppressing CD4^+^ T-cell proliferation on a per-cell basis.

### Caspase-8 deficiency alters macrophage TLR responses in vivo but has a minimal effect on myeloid cell homeostasis

**Fig. 3:**
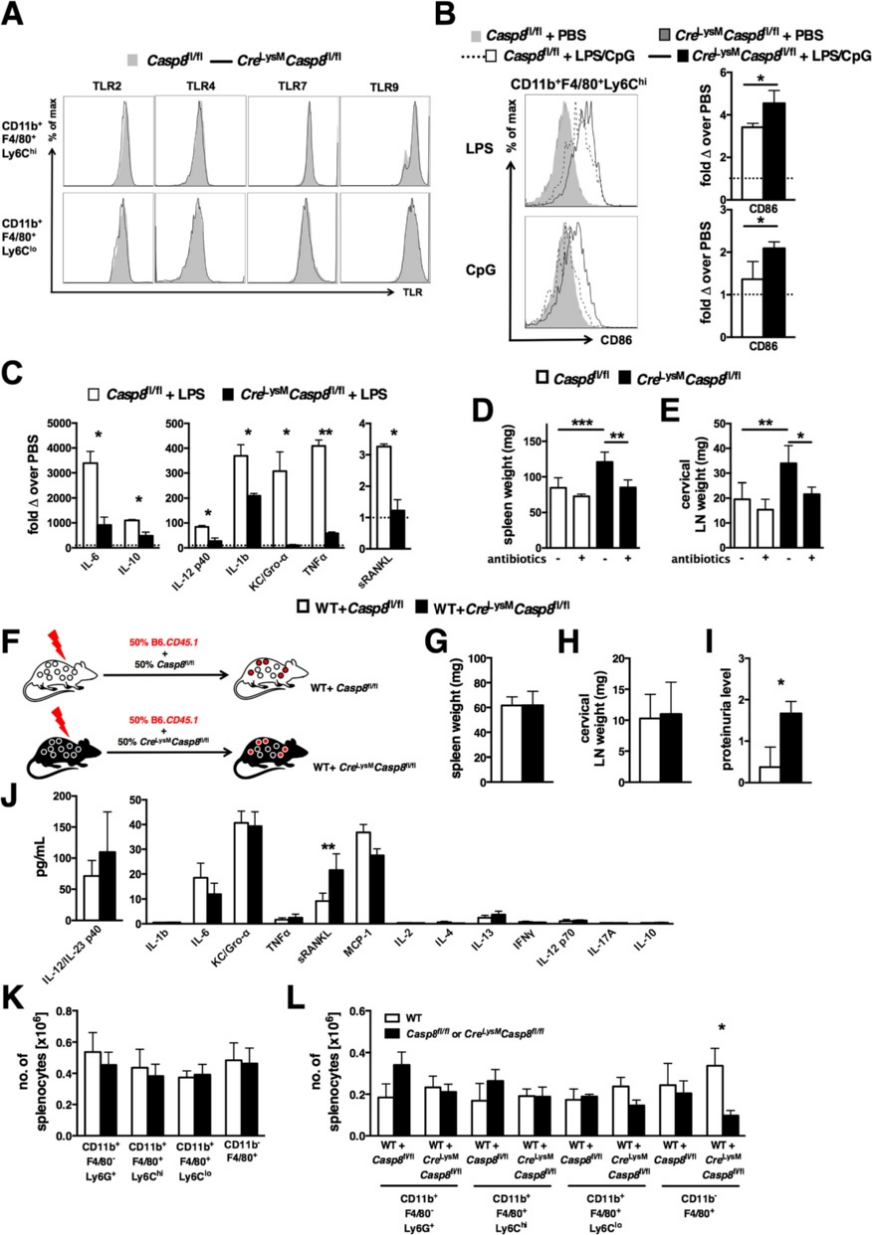
Caspase-8 deficiency alters the macrophage TLR response in vivo but does not affect cell survival. **a** Splenocytes from 6–8-month-old (aged) female *Casp8* ^fl/fl^ (control) and *Cre* ^LysM^ *Casp8* ^fl/fl^ mice (n ≥ 7) were analyzed by flow cytometry. Shown are representative fluorescence-activated cell sorting (FACS) plots of splenic CD11b^+^F4/80^+^Ly6C^high^ and CD11b^+^F4/80^+^Ly6C^low^ populations displaying relative levels of TLR expression. **b** and **c** Representative FACS plots and quantitative graphs of results representing the fold change in CD86 expression over PBS injection alone. **b** 3-month-old control and *Cre* ^LysM^ *Casp8* ^fl/fl^ mice (n = 4) that received LPS or CpG injection (200 μg/mouse) were evaluated 4 h later for splenic CD11b^+^F4/80^+^Ly6C^high^ cell expression of CD86. **c** Serum levels of cytokines and chemokines from TLR agonist–injected mice. **d** and **e** 3-week-old control and *Cre* ^LysM^ *Casp8* ^fl/fl^ mice (n = 4) treated with oral antibiotics (ampicillin, vancomycin, neomycin sulfate, metronidazole) for 8 weeks were evaluated for **d** spleen weight and **e** cervical lymph node weight. **f**–**l** Mice reconstituted with equal portions of B6.*CD45.1* (wild-type [WT]) and either control or *Cre* ^LysM^ *Casp8* ^fl/fl^ FACS-sorted LSK populations (n = 5) were maintained on low-dose oral antibiotics. **f** Representation of chimera generation. Chimeric mice were evaluated 8 months posttransfer for **g** splenomegaly, **h** lymphadenopathy, **i** proteinuria, **j** serum cytokine and chemokine levels, **k** myeloid cell subset numbers, and **l** distribution of WT (45.1)-derived and control or *Cre* ^LysM^ *Casp8* ^fl/fl^ (45.2)-derived myeloid populations. Data are represented as mean ± SD and were compared by Mann–Whitney *U* test: *p < 0.05; **p < 0.005; ***p < 0.0005. *TLR* Toll-like receptor, *Casp8* caspase-8, *LPS* lipopolysaccharide, *PBS* phosphate-buffered saline, *IL* interleukin, *Gro-α* growth-regulated oncogene-α, *IFN* interferon, *Ig* immunoglobulin, *KC* keratinocyte chemoattractant, *TNF* tumor necrosis factor, *sRANKL* soluble receptor activator of nuclear factor κB ligand, *LN* lymph node, *WT* wild type, *MCP* monocyte chemoattractant protein, *LSK* lineage-negative, Sca-1^+^, c-kit^+^

As TLRs play a central role in macrophage activation, we assessed the in vivo expression pattern of TLRs and response to TLR agonists of macrophages in the absence of caspase-8. TLR2, TLR4, TLR7, and TLR9 expression was similar between *Cre*^LysM^*Casp8*^fl/fl^ and control Ly6C^high^ and Ly6C^low^ splenic CD11b^+^F4/80^+^ cells (Fig. 3a). To determine the functional response of these TLRs in caspase-8–deficient populations, LPS, imiquimod, or CpG was intraperitoneally injected into *Cre*^LysM^*Casp8*^fl/fl^ and control mice. Both TLR4 and TLR9 in vivo activation induced increased CD86 expression on caspase-8–deficient Ly6C^high^ splenic CD11b^+^F4/80^+^ cells compared with control cells (Fig. 3b). These data suggest that, although this population expressed CD86 at normal levels under steady-state conditions, caspase-8 deficiency confers the capacity to upregulate this costimulatory marker upon TLR activation. Further, TLR4 and TLR9 in vivo activation induced elevated levels of circulating anti- and proinflammatory cytokines in *Cre*^LysM^*Casp8*^fl/fl^ and control mice (Fig. 3c and Additional file 1: Figure S3), but to a much greater extent in control mice in response to TLR4 activation (Fig. 3c). Over the past several years, gut microflora have been suggested to be a reservoir for endogenous TLR ligands [18, 19]. To reduce the potential for endogenous TLR ligands from gut microflora, young *Cre*^LysM^*Casp8*^fl/fl^ mice were treated with oral antibiotics, which prevented both splenomegaly (Fig. 3d) and lymphadenopathy (Fig. 3e) compared with untreated *Cre*^LysM^*Casp8*^fl/fl^ mice.

Further, the survival of myeloid cell subsets was examined using mixed bone marrow chimeric mice (Fig. 3f) maintained on low-dose oral antibiotics. As expected on the basis of our prior results with oral antibiotic treatment, mixed bone marrow chimeric mice (wild-type [WT] + *Cre*^LysM^*Casp8*^fl/fl^) did not display splenomegaly or lymphadenopathy (Fig. 3g, h). However, proteinuria persisted in these mice despite low-dose oral antibiotic treatment (Fig. 3i). Consistent with our observations in aged *Cre*^LysM^*Casp8*^fl/fl^ mice, serum levels of sRANKL were elevated in WT + *Cre*^LysM^*Casp8*^fl/fl^ mice compared with WT + *Casp8*^fl/fl^ mice (Fig. 3j). Loss of caspase-8 in myeloid cells did not result in enhanced survival, as splenic myeloid cell numbers remained unchanged in mixed bone marrow chimeric mice (Fig. 3k). Further, there was no preferential expansion of CD11b^+^*Cre*^LysM^*Casp8*^fl/fl^-derived splenic myeloid populations in mixed bone marrow chimeric mice (Fig. 3*l*). These data indicate that loss of caspase-8 in vivo does not confer a survival advantage to splenic myeloid cells. However, we observed that the *Cre*^LysM^*Casp8*^fl/fl^-derived CD11b^−^F4/80^+^ red pulp macrophage population exhibited reduced expansion in mixed bone marrow chimeric mice. Taken together, these data suggest that caspase-8 in myeloid cells controls the TLR response to the gut microflora and plays a only minor role in myeloid cell survival.

Because caspase-8 is a downstream signaling component of the DR Fas, the responses to cell death stimuli were evaluated in caspase-8–deficient myeloid cells. Total splenocytes from *Cre*^LysM^*Casp8*^fl/fl^ and control mice were incubated with either Fas ligand (FasL) or etoposide. Whereas ex vivo splenic Ly6C^high^ and Ly6C^low^ CD11b^+^F4/80^+^ cells were susceptible to both FasL-induced (Additional file 1: Figure S4a, b) and etoposide-induced (Additional file 1: Figure S4a, c) death, splenic neutrophils appeared responsive only to FasL. Thus, the availability of caspase-8 had no effect on the level of cell death in response to either treatment.

### RIPK3 deletion prevents inflammatory phenotypes in *Cre*^LysM^*Casp8*^fl/fl^ mice

**Fig. 4:**
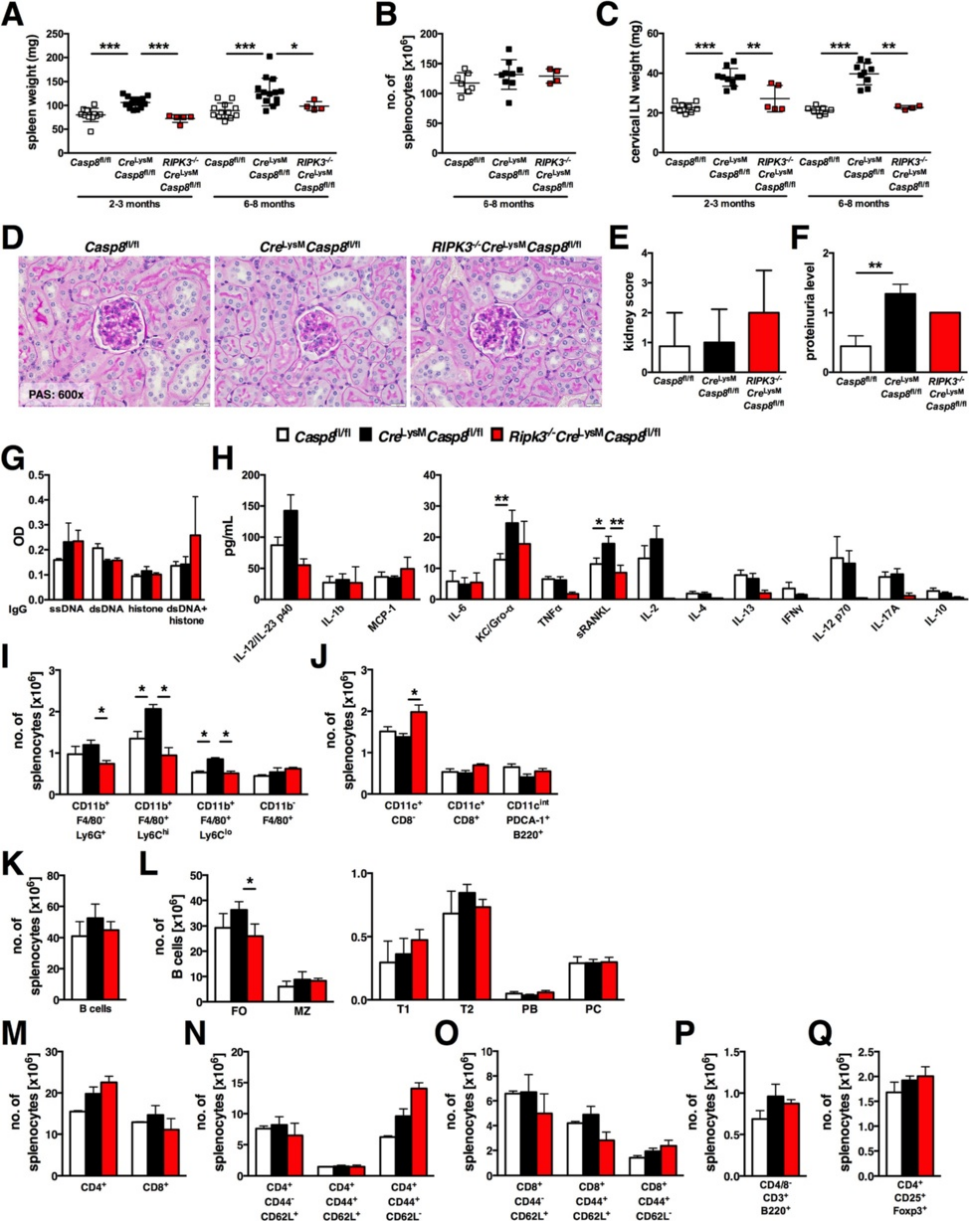
RIPK3 deletion prevents inflammation in *Cre* ^LysM^ *Casp8* ^fl/fl^ mice. 2–3-month-old (young) and 6–8-month-old (aged) female *Casp8* ^fl/fl^ (control), *Cre* ^LysM^ *Casp8* ^fl/fl^, and *RIPK3* ^−/−^ *Cre* ^LysM^ *Casp8* ^fl/fl^ mice (n ≥ 4) were evaluated for systemic autoimmune disease phenotypes, and splenocytes from aged mice were analyzed by flow cytometry. **a** Spleen weights of young and aged mice. **b** Total numbers of live splenocytes from aged mice. **c** Cervical lymph node weights of young and aged mice. **d** Formalin-fixed kidney sections (5 μm) from aged mice stained with periodic acid–Schiff (PAS). **e** Kidney scores of aged mice. **f** Proteinuria of aged mice assessed using Uristix reagent strips. Serum from aged mice was evaluated for levels of **g** ssDNA-, dsDNA-, histone-, and chromatin-reactive IgG antibodies and **h** cytokines and chemokines. **i** Number of CD11b^+^F4/80^−^Ly6G^+^ neutrophils, Ly6C^high^ and Ly6C^low^ CD11b^+^F4/80^+^ cells and CD11b^−^F4/80^+^ red pulp macrophages. **j** Number of CD11c^+^CD8^−^ and CD11c^+^CD8^−^ conventional DCs and CD11c^intermediate^PDCA-1^+^B220^+^ plasmacytoid DCs. **k** Total B-cell (CD11c^−^B220^+^) numbers. **l** B-cell subsets: follicular (FO; CD19^+^CD21/35^+^CD23^+^), marginal zone (MZ; CD19^+^CD21/35^+^CD23^low^), transitional 1 (T1; B220^+^AA4.1^+^CD23^−^), transitional 2 (T2; B220^+^AA4.1^+^CD23^+^), plasmablasts (PB; CD19^+^B220^low^CD138^+^CD21/35^−^CD23^−^), and plasma cells (PC; CD19^+^B220^+^CD138^+^CD21/35^−^CD23^−^). **m** Total CD4^+^ and CD8^+^ T-cell numbers. Naïve (CD44^−^CD62L^+^), central memory (CD44^+^CD62L^+^), and activated (CD44^+^CD62L^−^) **n** CD4^+^ and **o** CD8^+^ T-cell numbers. **p** CD4^−^CD8^−^CD3^+^B220^+^ double-negative T-cell numbers. **q** CD4^+^CD25^+^Foxp3^+^ regulatory T-cell numbers. **a**–**h** represent analyses that included data from control and *Cre* ^LysM^ *Casp8* ^fl/fl^ mice shown in Figs. 1b–d, f–h, and j. Data are represented as mean ± SD and were compared by Mann–Whitney *U* test: *p < 0.05; **p < 0.005; ***p < 0.0005. *Casp8* caspase-8, *LPS* lipopolysaccharide, *IL* interleukin, *Gro-α* growth-regulated oncogene-α, *IFN* interferon, *Ig* immunoglobulin, *KC* keratinocyte chemoattractant, *TNF* tumor necrosis factor, *MCP* monocyte chemoattractant protein, *LN*, lymph node, *OD* optical density, *RIPK* receptor-interacting serine/threonine protein kinase, *PDCA-1* plasmacytoid dendritic cell antigen 1, *sRANKL* soluble receptor activator of nuclear factor κB ligand

Owing to involvement of caspase-8 in limiting RIPK3 activity [2], we generated *RIPK3*^−/−^*Cre*^LysM^*Casp8*^fl/fl^ mice. Deletion of RIPK3 prevented inflammatory disease phenotypes observed in *Cre*^LysM^*Casp8*^fl/fl^ mice. Splenomegaly was reduced in both young and aged *RIPK3*^−/−^*Cre*^LysM^*Casp8*^fl/fl^ mice compared with *Cre*^LysM^*Casp8*^fl/fl^ mice (Fig. 4a), with no alteration in total splenocyte numbers (Fig. 4b). Lymphadenopathy was also abated in both young and aged *RIPK3*^−/−^*Cre*^LysM^*Casp8*^fl/fl^ mice compared with *Cre*^LysM^*Casp8*^fl/fl^ mice (Fig. 4c). Similar to control and *Cre*^LysM^*Casp8*^fl/fl^ mice, *RIPK3*^−/−^*Cre*^LysM^*Casp8*^fl/fl^ mice showed little evidence of kidney damage (Fig. 4d, e). However, a trend toward reduced proteinuria levels (Fig. 4f) was detected in *RIPK3*^−/−^*Cre*^LysM^*Casp8*^fl/fl^ mice compared with *Cre*^LysM^*Casp8*^fl/fl^ mice. Similar to *Cre*^LysM^*Casp8*^fl/fl^ mice, there was no evidence of circulating ssDNA, dsDNA, or histone IgG antibodies in *RIPK3*^−/−^*Cre*^LysM^*Casp8*^fl/fl^ mice (Fig. 4g). However, there was a trend toward increased dsDNA + histone IgG antibodies in serum from *RIPK3*^−/−^*Cre*^LysM^*Casp8*^fl/fl^ mice compared with control and *Cre*^LysM^*Casp8*^fl/fl^ mice (Fig. 4g). Further, elevated serum levels of sRANKL in *Cre*^LysM^*Casp8*^fl/fl^ mice (Fig. 2) were reversed with deletion of RIPK3. Moreover, the increased numbers of splenic Ly6C^high^CD11b^+^F4/80^+^, Ly6C^low^CD11b^+^F4/80^+^, and Ly6G^+^ (neutrophils) cells in *Cre*^LysM^*Casp8*^fl/fl^ mice were restored to control levels by deletion of RIPK3 (Fig. 4i). Although *Cre*^LysM^*Casp8*^fl/fl^ mice showed no statistical difference in DC numbers compared with control mice, *RIPK3*^−/−^*Cre*^LysM^*Casp8*^fl/fl^ mice exhibited increased numbers of conventional DCs compared with *Cre*^LysM^*Casp8*^fl/fl^ mice (Fig. 4j). Additionally, whereas total B-cell numbers were unaffected in *RIPK3*^−/−^*Cre*^LysM^*Casp8*^fl/fl^ mice (Fig. 4k), there was a decrease in the CD19^+^CD21/35^+^CD23^+^ follicular B-cell compartment in *RIPK3*^−/−^*Cre*^LysM^*Casp8*^fl/fl^ mice compared with *Cre*^LysM^*Casp8*^fl/fl^ mice (Fig. 4*l*). However, in *Cre*^LysM^*Casp8*^fl/fl^ mice, deletion of RIPK3 did not affect numbers of total CD4^+^ or CD8^+^ (Fig. 4m), naïve or activated CD4^+^ (Fig. 4n) or CD8^+^ (Fig. 4o), double-negative (Fig. 4p), or regulatory T cells (Fig. 4q). These results suggest that the inflammation observed in *Cre*^LysM^*Casp8*^fl/fl^ mice arises in part from unchecked RIPK3 activity, as deletion of RIPK3 can restore these aberrant phenotypes to normal.

Previous studies in lymphocytes showed that loss of caspase-8 results in necroptosis, which is mediated by RIPK1/RIPK3 signaling. Therefore, the role that caspase-8 plays in macrophage death was evaluated in vitro. M-CSF-generated caspase-8–deficient BMDMs expressed Fas under steady-state conditions, which was upregulated with TLR activation (Additional file 1: Figure S5). Without stimulation, BMDMs deficient in caspase-8, RIPK3, or both caspase-8 and RIPK3 displayed increased LDH activity, indicating an increase in cell death. Similar to ex vivo caspase-8–deficient myeloid populations, caspase-8–deficient BMDMs responded like control BMDMs to FasL and etoposide at 48 h (Additional file 1: Figure S6) posttreatment, despite the overall higher cell death in caspase-8–deficient BMDM cultures. However, unlike in control BMDMs, the addition of Z-VAD did not reverse the death induced by FasL in caspase-8–deficient cells (Additional file 1: Figure S6), indicating that these caspase-8–deficient BMDMs underwent a caspase-independent cell death. Deletion of RIPK3 in caspase-8–deficient BMDMs restored the response to that of control BMDMs, as the addition of Z-VAD blocked death in *RIPK3*^−/−^*Cre*^LysM^*Casp8*^fl/fl^ BMDMs. Taken together, these results suggest that caspase-8 deficiency predisposes macrophages to caspase-independent cell death upon DR ligation.

### Caspase-8 deficiency in macrophages alters the response to TLR activation and macrophage polarization in vitro

To expand upon these studies, caspase-8–deficient BMDMs were treated with LPS, imiquimod, or CpG in the presence of absence of necrostatin-1 (Nec-1) and were evaluated for cytokine transcript levels as well as levels of cytokines in supernatants at indicated time points. Caspase-8–deficient BMDMs showed reduced transcription of IL-10 upon TLR7 and TLR9 ligation and reduced IL-12/IL-23p40 transcription with TLR4, TLR7, and TLR9 activation compared with control BMDMs. However, IL-10 transcript was elevated in caspase-8–deficient BMDMs upon TLR4 stimulation (Additional file 1: Figure S7a, b). Similar to transcription levels, caspase-8–deficient BMDMs produced less IL-10 and IL-12/IL-23p40 with TLR4, TLR7, and TLR9 ligation compared with control BMDMs (Additional file 1: Figure S7a, b). However, both transcript and protein levels of IL-10 and IL-12/IL-23p40 were restored to control levels with RIPK3 deletion, but not with blockade of RIPK1 kinase activity via the addition of Nec-1 (Additional file 1: Figure S7a, b).

**Fig. 5:**
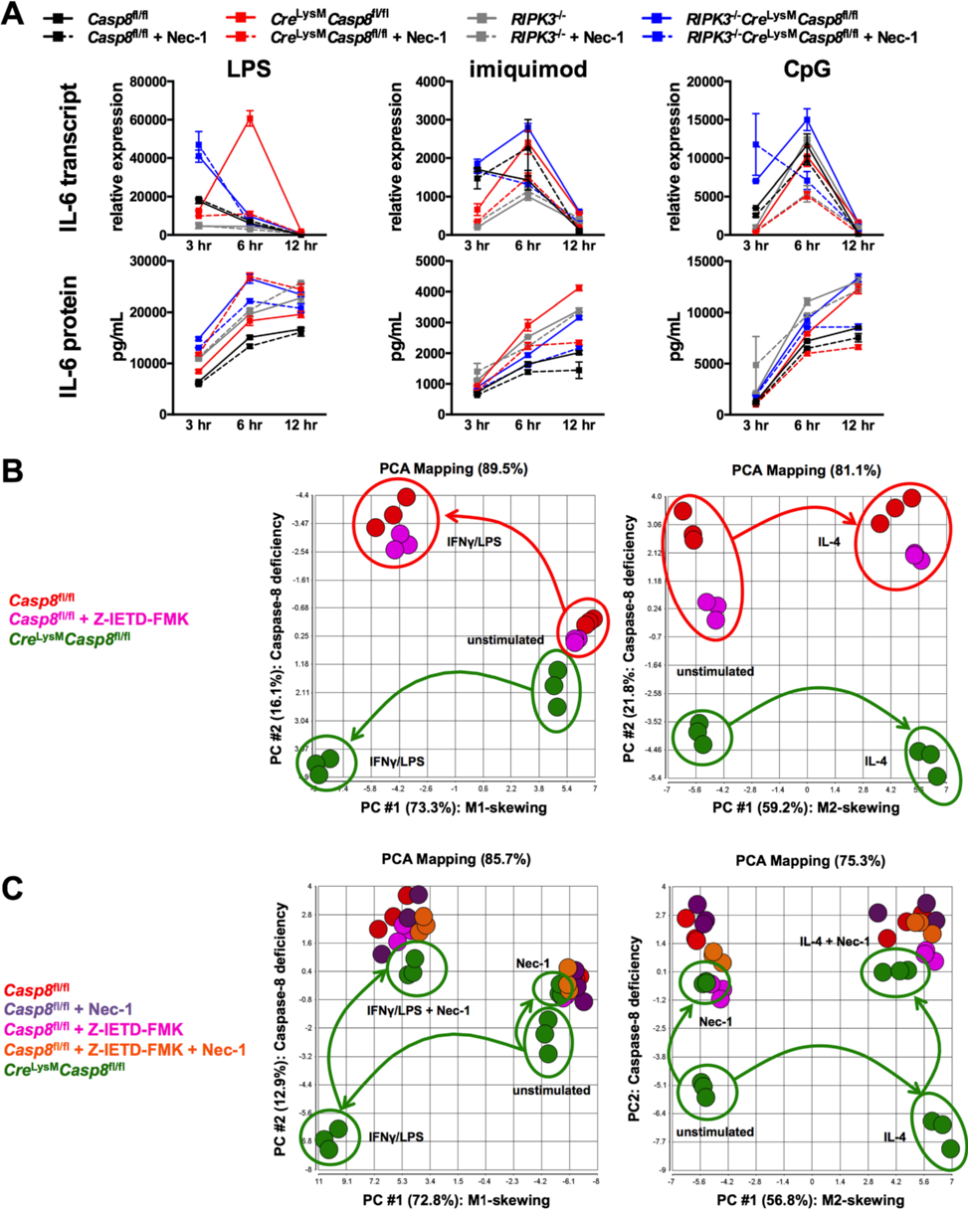
Caspase-8 deficiency in macrophages alters the response to Toll-like receptor activation and macrophage polarization in vitro. **a** *Casp8* ^fl/fl^ (control), *Cre* ^LysM^ *Casp8* ^fl/fl^, *RIPK3* ^−/−^, and *RIPK3* ^−/−^ *Cre* ^LysM^ *Casp8* ^fl/fl^ BMDMs were stimulated with LPS (10 ng/ml), imiquimod (5 μg/ml), and CpG (5 μg/ml) with or without Nec-1 (30 μM) for 3, 6, and 12 h and evaluated for transcript and supernatant levels of IL-6. Data are represented as mean ± SD of biological triplicates, and experiments were repeated twice. **b** and **c** Control, control + Z-IETD-FMK (20 μM), and *Cre* ^LysM^ *Casp8* ^fl/fl^ BMDMs were cultured with M1-polarizing conditions (primed overnight with IFN-γ 100 ng/ml and stimulated for 3 h with LPS 10 ng/ml) and M2-polarizing conditions (stimulated for 24 h with IL-4 40 ng/ml) with or without Nec-1. Principal component analysis of gene expression was performed in BMDMs under (**b**) M1- and M2-polarizing conditions and (**c**) M1- and M2-polarizing conditions + Nec-1. Data are represented as biological triplicates. *FMK* fluoromethylketone, *IFN* interferon, *IL* interleukin, *LPS* lipopolysaccharide, *Nec-1* necrostatin-1, *PCA* principal component analysis, *Z-IETD* Z-Ile-Glu-(O-ME)-Thr-Asp-(O-Me)

Whereas IL-6 and TNF-α transcription was increased with TLR4 and TLR7 activation in caspase-8–deficient BMDMs, these BMDMs produced higher levels of IL-6 and TNF-α in response to TLR4, TLR7, and TLR9 ligation compared with control BMDMs (Fig. 5a and Additional file 1: Figure S7c). Transcription of IL-6 was reduced with Nec-1 in response to TLR4, TLR7, and TLR9 ligation and with RIPK3 deletion in response to TLR4 activation (Fig. 5a). In addition, RIPK1 blockade reduced transcription of TNF-α in caspase-8–deficient BMDMs following stimulation with TLR4 and TLR7 (Additional file 1: Figure S7c). Similarly to transcription, blockade of RIPK1 reduced production of TNF-α by *Cre*^LysM^*Casp8*^fl/fl^ BMDMs in response to TLR4, TLR7, and TLR9 activation. However, suppression of RIPK1 affected only IL-6 following TLR7 and TLR9 activation. In contrast, deletion of RIPK3 reduced TNF-α production by *Cre*^LysM^*Casp8*^fl/fl^ BMDMs in response to TLR7 and TLR9 activation and IL-6 in response to TLR7 activation (Fig. 5a and Additional file 1: Figure S7C). Elevated transcription and hypersecretion of IL-1β without the requirement for ATP was observed with TLR4, TLR7, and TLR9 ligation in *Cre*^LysM^*Casp8*^fl/fl^ BMDMs and was also blocked by the addition of Nec-1 or by RIPK3 deletion (Additional file 1: Figure S7D). Further, IL-1β was secreted at increased levels, and abrogated by Nec-1, in *Cre*^LysM^*Casp8*^fl/fl^ BMDMs compared with control BMDMs following stimulation with TLR4, TLR7, and TLR9 and addition of ATP (Additional file 1: Figure S7E).

The environmental milieu has been shown to influence macrophages to assume alternate phenotypes. We examined whether caspase-8 was involved in the polarization of BMDMs to classically activated M1 macrophages that are proinflammatory or to alternatively activated M2 macrophages that are necessary for repair. Polarization of BMDMs was analyzed via gene expression using a custom QuantiGene 2.0 panel [20] (see Additional file 1: Table S1) following stimulation with IFN-γ/LPS (M1-skewing media) and IL-4 (M2-skewing media). Among the 52 genes included in the panel, those genes that were differentially expressed between populations of BMDMs according to selected criteria (differentially expressed in at least one two-group comparison with Bonferroni-corrected *p* value <0.05) can be found in Additional file 2. Further, heat maps were generated to visualize differential gene expression between unstimulated control and caspase-8–deficient BMDMs (Additional file 1: Figure S8A), *Cre*^LysM^*Casp8*^fl/fl^ BMDMs with and without Nec-1 (Additional file 1: Figure S8B), M1-polarized control and *Cre*^LysM^*Casp8*^fl/fl^ BMDMs (Additional file 1: Figure S8C), unstimulated *Cre*^LysM^*Casp8*^fl/fl^ and M1-polarized *Cre*^LysM^*Casp8*^fl/fl^ BMDMs (Additional file 1: Figure S8D), M1-polarized *Cre*^LysM^*Casp8*^fl/fl^ with and without Nec-1 (Additional file 1: Figure S8E), M2-polarized control and *Cre*^LysM^*Casp8*^fl/fl^ BMDMs (Additional file 1: Figure S8F), unstimulated *Cre*^LysM^*Casp8*^fl/fl^ and M2-polarized *Cre*^LysM^*Casp8*^fl/fl^ BMDMs (Additional file 1: Figure S8G), and M2-polarized *Cre*^LysM^*Casp8*^fl/fl^ with and without Nec-1 (Additional file 1: Figure S8H). PCA revealed that unstimulated caspase-8–deficient BMDMs cluster separately from control BMDMs, whereas BMDMs incubated with the inhibitor of caspase-8 enzymatic activity, Z-IETD-FMK, cluster more closely with control BMDMs (Fig. 5b). Further, *Casp8*^fl/fl^, *Casp8*^fl/fl^ + Z-IETD-FMK, and *Cre*^LysM^*Casp8*^fl/fl^ BMDMs behaved similarly in response to M2-skewing media (Fig. 5b). However, *Cre*^LysM^*Casp8*^fl/fl^ BMDMs clustered independently from both *Casp8*^fl/fl^ and *Casp8*^fl/fl^ + Z-IETD-FMK BMDMs in response to M1-skewing media (Fig. 5b). In all instances, the addition of Nec-1 restored caspase-8–deficient BMDM populations to those of control BMDMs (Fig. 5c). These data suggest not only that caspase-8 is involved in the suppression of macrophage responses to TLR activation in an RIPK1- and RIPK3-dependent manner, but also that caspase-8 controls macrophage polarization in response to M1-skewing media in an RIPK1-dependent fashion.

## Discussion

Our data suggest that caspase-8 controls the response of macrophages to TLR activation and M1-skewing media by dampening RIPK activity. *Cre*^LysM^*Casp8*^fl/fl^ mice develop a mild systemic inflammatory disease characterized by splenomegaly, lymphadenopathy, immune complex deposition in the kidney, proteinuria, and elevated amounts of serum cytokines and antibodies. The observed splenomegaly is not attributable to increased numbers of splenocytes, indicating that the spleen may be enlarged for other reasons, such as increased red blood cell numbers or elevated collagen deposition. Although we observed increased Ly6C^high^ and Ly6C^low^ CD11b^+^F4/80^+^ splenic populations, analysis of mixed bone marrow chimeric mice reveals that these caspase-8–deficient populations are not preferentially expanded, indicating that perhaps disease progression, rather than lack of caspase-8, increases these populations, potentially through increased migration, decreased egress, and/or enhanced proliferation of progenitor populations. Further, the systemic inflammatory phenotypes in *Cre*^LysM^*Casp8*^fl/fl^ mice may arise independently of the role of caspase-8 in survival, as mixed bone marrow chimeric mice are reconstituted with equal proportions of normal and caspase-8–deficient splenic populations. Moreover, deletion of RIPK3 is sufficient to prevent inflammation in *Cre*^LysM^*Casp8*^fl/fl^ mice and restore the CD11b^+^F4/80^+^ splenic populations to those of control mice. The increased CD11b^+^F4/80^+^ splenic populations indicate that the absence of caspase-8 does not lead to rampant necroptosis due to RIPK3 action, as is the case with T-cell caspase-8 deficiency [21]. Further, if insufficient apoptosis due to the lack of caspase-8 was the cause of increased CD11b^+^F4/80^+^ splenic populations, deletion of RIPK3 would exacerbate rather than correct this phenotype, as necroptosis would also be prevented. Rather, these data suggest that caspase-8 functions to suppress RIPK activity in myeloid populations independent of their roles in cell death.

Upon TLR stimulation, caspase-8–deficient BMDMs produce elevated proinflammatory cytokines, in part due to rampant RIPK1 and RIPK3 activity, as the addition of Nec-1 or RIPK3 deletion blocks production. Further, compared with controls, *Cre*^LysM^*Casp8*^fl/fl^ mice show an expansion of costimulatory CD86 expression on Ly6C^high^CD11b^+^F4/80^+^ splenocytes upon injection with TLR4 and TLR9 agonists. These results are similar to those observed in *Cre*^CD11c^*Casp8*^fl/fl^ mice, in that caspase-8–deficient BMDCs also produce elevated levels of proinflammatory cytokines and respond to in vivo TLR7 activation by hyperactivation of splenic CD11c^+^CD8^−^ DCs compared with controls [10]. However, caspase-8 function diverges in myeloid cells and DCs upon elimination of the gut microflora. The gut microflora are a means to maintain homeostasis in the gut, but these microbes have also been suggested to be a reservoir of endogenous TLR ligands [19]. When the immune system is compromised, as with the case of caspase-8 deletion, the response to these microbial pathogen-associated molecular patterns (PAMPs) may go awry. Whereas treatment with oral antibiotics is ineffective at reducing the systemic inflammation in *Cre*^CD11c^*Casp8*^fl/fl^ mice [10], this same treatment prevents splenomegaly and lymphadenopathy in *Cre*^LysM^*Casp8*^fl/fl^ mice. These results suggest that caspase-8 signals via mechanisms in myeloid cells different from those in DCs and that caspase-8 in myeloid populations potentially controls the response to endogenous gut microflora. Further, the impact of oral antibiotic treatment mimics the effect of RIPK3 deletion in *Cre*^LysM^*Casp8*^fl/fl^ mice, indicating a potential involvement of RIPK3 in the maintenance of tolerance to gut microflora. An alternate explanation could be that necroptotic macrophages in the gut release damage-associated molecular patterns (DAMPs) capable of activating other cells previously “primed” by gut microflora [22]. Removal of the gut microflora would prevent this priming, thus inhibiting activation of the immune system by DAMPs released by necroptotic macrophages and systemic inflammation.

Conditional deletion has revealed roles for caspase-8 in a number of cell death–independent activities. TLR4, TLR7, and TLR9 activation induces hyperproduction of proinflammatory cytokines in caspase-8–deficient BMDMs. Similar to caspase-8–deficient DCs [10], blocking RIPK1 kinase activity dampens the TLR-induced secretion of proinflammatory cytokines in caspase-8–deficient macrophages. TLR engagement can induce RIPK signaling independent of DR activation, thereby leading to formation of a ripoptosome, a complex containing proteins that participate in necroptosis, including RIPK1, caspase-8, and cFLIP [8, 23]. Recent evidence suggests that ripoptosome activity and RIPK3 signaling in BMDMs can induce production of proinflammatory cytokine IL-1β in a caspase-8–dependent manner [6] independent of cell death. Both caspase-8–deficient BMDCs [10] and BMDMs secrete elevated IL-1β in response to TLR activation with the addition of ATP. Nec-1 decreases this IL-1β secretion in both control and casapse-8–deficient BMDCs [10] and BMDMs, albeit to a lesser extent, indicating that RIPK1 is potentially involved in IL-1β release independent of caspase-8. In addition, we and others have shown that caspase-8–deficient BMDCs activated by LPS secrete IL-1β without the requirement for secondary ATP stimulation [5, 10]. Further, we previously reported that this secretion of IL-1β by BMDCs also occurs via TLR7 and TLR9 activation [10]. Similar to our previous findings with BMDCs, we show that caspase-8–deficient BMDMs secrete IL-1β without the requirement for secondary ATP stimulation in response to TLR4, TLR7, and TLR9 activation and that this secretion is abrogated by the addition of Nec-1 and deletion of RIPK3. Thus, our data implicate caspase-8 in the suppression of the macrophage TLR response in a manner that may require the components of the ripoptosome.

SLE is a severe multisystem autoimmune disease characterized by an immune response mounted against nuclear self-antigens that results in multiple tissue and/or organ damage. Loss of Fas in myeloid cells [9] or loss of caspase-8 in DCs [10] results in a SLE-like disease. Although *Cre*^LysM^*Casp8*^fl/fl^ mice present with hallmarks of SLE-like disease, these phenotypes are markedly less robust than those observed in *Cre*^LysM^*Fas*^fl/fl^ or *Cre*^CD11c^*Casp8*^fl/fl^ mice. Similar to *Cre*^LysM^*Casp8*^fl/fl^ mice, *Cre*^LysM^*FADD*^fl/fl^ mice develop a mild systemic inflammation that is reversed by RIPK3 deletion [24]. These results indicate that Fas, FADD, and caspase-8 play a cell-type–specific role in suppressing autoimmune phenotypes. Although *Cre*^LysM^*Fas*^fl/fl,^*Cre*^LysM^*FADD*^fl/fl^ and *Cre*^LysM^*Casp8*^fl/fl^ myeloid populations are numerically dysregulated, their activation status is not altered. In contrast, *Cre*^CD11c^*Casp8*^fl/fl^ DC populations display an elevated expression of costimulatory molecules. Further, the splenomegaly observed in *Cre*^LysM^*Casp8*^fl/fl^ does not result in increased lymphocyte populations, which is different from the situation in *Cre*^LysM^*Fas*^fl/fl^ and *Cre*^LysM^*FADD*^fl/fl^ mice [9, 24]. However, both caspase-8– and Fas-deficient macrophages are unable to directly induce T-cell proliferation in mixed leukocyte reactions. Thus, these data suggest that, in myeloid cell–specific caspase-8– or Fas-deficient mice, the effect on lymphocytes is indirect and may be mediated by cytokines or by a macrophage-specific paracrine effect on nonlymphocytic cells such as DCs.

Investigators in previous studies have examined the effect of myeloid cell–specific caspase-8 deletion [5, 14]. In contrast to the data presented here, there was a failure to produce M-CSF-stimulated BMDMs from myeloid cell–specific caspase-8–deficient bone marrow. Additionally, there was no in vivo dysregulation of macrophage populations. These previous findings conflict with the present studies in that *Cre*^LysM^*Casp8*^fl/fl^ mice present increased numbers of both Ly6C^high^ and Ly6C^low^ splenic CD11b^+^F4/80^+^ cells. Further, previous studies did not show caspase-8 deletion in peritoneal neutrophils, which is in contrast to the complete deletion of caspase-8 in *Cre*^LysM^*Casp8*^fl/fl^ splenic neutrophils. The conflicting results observed between the *Cre*^LysM^*Casp8*^fl/fl^ mice and previously published strains presumably stem from differences in the generation of in vitro bone marrow–derived populations and the cell-specific caspase-8 deletion constructs. In the present study, BMDMs were generated with M-CSF, whereas researchers in previous studies relied on the use of L929 media, which can contain unpredictable levels of M-CSF as well as other unknown components. Additionally, in the present study, both alleles of caspase-8 are floxed, whereas previous studies floxed only one allele of caspase-8 and the other was deleted [14]. Because caspase-8 has been shown to be necessary for early myeloid progenitor formation [25, 26], deletion of caspase-8 in mice that have only one allele of caspase-8 may increase selective pressure on these cells. Thus, in this scenario, the cells that have complete deletion will die before becoming monocytes or macrophages, whereas others that fail to delete caspase-8 live and are not selected. However, in mice with two alleles of floxed caspase-8, progenitor deletion of caspase-8 may not be complete but caspase-8 is fully deleted as cells mature (Additional file 1: Figure S1), thereby allowing for cell survival throughout the differentiation process.

## Conclusions

In *Cre*^LysM^*Casp8*^fl/fl^ mice, activation of TLRs, potentially via PAMPs derived from gut microflora, may lead to increased RIPK1 and RIPK3 activity, induction of costimulatory molecules, and increased production of proinflammatory cytokines, ultimately culminating in a mild systemic inflammatory disease. Our data suggest that, under steady-state conditions, following TLR activation of myeloid cells by the gut microflora, caspase-8 associates with RIPK1 and RIPK3 and limits their downstream signaling, thereby preventing the continued activation of these cells to keep systemic inflammation in check. These data provide a macrophage-specific link between caspase-8 and the heightened TLR responses to endogenous ligands leading to inflammation and show, for the first time to our knowledge, that caspase-8 controls the macrophage response to TLR activation and polarization.

## Additional files

Additional file 1: Table S1. Affymetrix QuantiGene 2.0 custom panel 21522 for analysis of macrophage polarization. **Figure S1.** Genotype validation of splenocyte populations. **Figure S2.** Genotype validation of BMDMs. **Figure S3.** TLR9 in vivo activation induces upregulation of serum cytokines and chemokines at similar levels in *Casp8* ^fl/fl^ and *Cre* ^LysM^ *Casp8* ^fl/fl^ mice. **Figure S4.** Caspase-8–deficient splenic myeloid populations are not predisposed to aberrant death. **Figure S5.** Caspase-8–deficient BMDMs express Fas. **Figure S6.** Caspase-8–deficient BMDMs undergo caspase-independent cell death in response to apoptotic stimuli. **Figure S7.** Caspase-8 deficiency in macrophages alters the response to TLR activation in vitro. **Figure S8.** Caspase-8 deficiency in macrophages alters the genetic profile in response to macrophage polarization in vitro. (PDF 5409 kb) Additional file 2: Table S2. ANOVA comparison data from Affymetrix QuantiGene 2.0 custom panel 21522 for analysis of macrophage polarization. (XLS 68 kb)

## Abbreviations

ANOVA: analysis of variance
APC: antigen-presenting cell
B6: C57BL/6
BMDC: bone marrow–derived dendritic cell
BMDM: bone marrow–derived macrophage
Casp8: caspase-8
cFLIP: cellular Fas-associated death domain protein–like interleukin-1β–converting enzyme-inhibitory protein
CFSE: carboxyfluorescein diacetate succinimidyl ester
DAMP: damage-associated molecular pattern
DC: dendritic cell
DR: death receptor
dsDNA: double-stranded DNA
FACS: fluorescence-activated cell sorting
FADD: Fas-associated death domain protein
FasL: Fas ligand
FMK: fluoromethylketone
FO: follicular
Gro-α: growth-regulated oncogene-α
HRP: horseradish peroxidase
IFN-γ: interferon γ
Ig: immunoglobulin
IL: interleukin
KC: keratinocyte chemoattractant
LDH: lactate dehydrogenase
LN: lymph node
LPS: lipopolysaccharide
LSK: lineage-negative, Sca-1^+^, c-kit^+^
MCP: monocyte chemoattractant protein
M-CSF: macrophage colony-stimulating factor
MZ: marginal zone
Nec-1: necrostatin-1
OD: optical density
OVA: ovalbumin
PAMP: pathogen-associated molecular pattern
PAS: periodic acid–Schiff
PB: plasmablast
PBS: phosphate-buffered saline
PC: plasma cell
PCA: principal component analysis
PDCA-1: plasmacytoid dendritic cell antigen 1
RIPK: receptor-interacting serine/threonine protein kinase
Sca-1: stem cell antigen 1
SD: standard deviation
SLE: systemic lupus erythematosus
sRANKL: soluble receptor activator of nuclear factor κB ligand
ssDNA: single-stranded DNA
T1: transitional 1
T2: transitional 2
TLR: Toll-like receptor
TNF: tumor necrosis factor
WT: wild type
Z-IETD: Z-Ile-Glu-(O-ME)-Thr-Asp-(O-Me)
Z-VAD: carbobenzoxy-valyl-alanyl-aspartyl-[*O*-methyl]
