# Supplementary material for: Conditional deletion of caspase-8 in macrophages alters macrophage activation in a RIPK-dependent manner
Source: Arthritis Res Ther. 2015 Oct 16;17:291. doi: 10.1186/s13075-015-0794-z (PMC4608154; doi:10.1186/s13075-015-0794-z)
Supplement: Additional file 1: Table S1. — Affymetrix QuantiGene 2.0 custom panel 21522 for analysis of macrophage polarization. Figure S1. Genotype validation of splenocyte populations. Figure S2. Genotype validation of BMDMs. Figure S3. TLR9 in vivo activation induces upregulation of serum cytokines and chemokines at similar levels in Casp8 fl/fl and Cre LysM Casp8 fl/fl mice. Figure S4. Caspase-8–deficient splenic myeloid populations are not predisposed to aberrant death. Figure S5. Caspase-8–deficient BMDMs express Fas. Figure S6. Caspase-8–deficient BMDMs undergo caspase-independent cell death in response to apoptotic stimuli. Figure S7. Caspase-8 deficiency in macrophages alters the response to TLR activation in vitro. Figure S8. Caspase-8 deficiency in macrophages alters the genetic profile in response to macrophage polarization in vitro. (PDF 5409 kb) [file 13075_2015_794_MOESM1_ESM.pdf]

Conditional deletion of caspase-8 in macrophages alters macrophage activation in a RIPK-dependent manner

Carla M. Cuda, PhD, Alexander V. Misharin, MD/PhD, Sonal Khare, PhD, Rana Saber, MS, FuNien Tsai, BS, Amy M. Archer, MD/PhD, Philip J. Homan, PhD, G. Kenneth Haines III, MD, Jack Hutcheson, PhD, Andrea Dorfleutner, PhD, G.R Scott Budinger, MD, Christian Stehlik, PhD and Harris Perlman, PhD

Table 1: Affymetrix QuantiGene 2.0 custom panel #21522 for analysis of macrophage polarization

	Protein	Gene	GeneBank Accession #	Role
1	NO synthetase	*Nos2*	NM_010927	M1
2	RELMα	*Retnla*	NM_020509	M2
3	Ym-1	*Chi3l3*	NM_009892	M2
4	Arginase	*Arg1*	NM_007482	M2
5	Tissue Transglutaminase 2	*Tgm2*	NM_009373	M2
6	Transferrin Receptor (CD71)	*Tfrc*	NM_011638	M2
7	Sphingosine kinase 1	*Sphk1*	NM_011451	M2
8	Tumor necrosis factor (ligand) superfamily, member 14	*Tnfsf14*	NM_019418	M2
9	Heparin-binding EGF-like growth factor	*Hbegf*	NM_010415	M2
10	FcyRIIb (CD32b)	*Fcgr2b*	NM_010187	M2
11	FcyRI (CD64)	*Fcgr1*	NM_010186.5	M1
12	CD163	*Cd163*	NM_053094	M2
13	CD36	*Cd36*	NM_007643	M2
14	Mannose Receptor (CD206)	*Mrc1*	NM_008625	M2
15	IL-12-p40	*IL12b*	NM_008352	M1
16	IL-12-p70	*IL12a*	NM_008351	M1
17	IL-10	*Il10*	NM_010548	M2
18	TGFbeta1	*Tgfb1*	NM_011577	M2
19	CXCL13	*Cxcl13*	NM_018866	M2
20	IL-6	*Il6*	NM_031168	M1
21	IL-23 (p19)	*Il23a*	NM_031252	M1
22	IL-1 beta	*Il1b*	NM_008361	M1
23	TNF	*Tnf*	NM_013693	M1
24	SOCS1	*Socs1*	NM_009896	M2
25	SOCS3	*Socs3*	NM_007707	M2
26	Nur77	*Nr4a1*	NM_010444	M2
27	JMJD3	*Kdm6b*	NM_001017426	M2
28	IRF4	*Irf4*	NM_013674	M2
29	IRF5	*Irf5*	NM_012057	M1
30	IRF8	*Irf8*	NM_008320	M1

31	PPARγ	*Pparg*	NM_011146	M2
32	SHIP	*Inpp5d*	NM_010566	M2
33	STAT6	*Stat6*	NM_009284	M2
34	P21	*Cdkn1a*	NM_007669	
35	KLF4	*Klf4*	NM_010637	M2
36	CARKL	*Shpk*	NM_029031	M2
37	NF-kB1 (p50)	*Nfkb1*	NM_008689	M1
38	C/EBPbeta	*Cebpb*	NM_009883	M2
39	Akt1	*Akt1*	NM_001165894	M2
40	Akt2	*Akt2*	NM_001110208	M1
41	IL-4R (CD124)	*Il4ra*	NM_001008700	M2
42	M-CSF (CD115)	*CSF1R*	NM_001037859	
43	GM-CSFR (CD116)	*CSF2RA*	NM_009970	
44	Tim-4	*Timd4*	NM_178759	M2
45	MerTK	*Mertk*	NM_008587	M2
46	PTEN	*Pten*	NM_008960	M1
47	IL-1RA	*Il1rn*	NM_001039701	M2
48	CD80	*CD80*	NM_009855	M1
49	CD86	*CD86*	NM_019388	M2
50	CCL17	*Ccl17*	NM_011332	M2
51	IL-33	*Il33*	NM_001164724	M2
52	CD39	*Entpd1*	NM_009848	M2
53	peptidylprolyl isomerase B	*Ppib*	NM_011149	housekeeping
54	ATPase, H+ transporting, lysosomal V1 subunit A	*Atp6v1a*	NM_007508	housekeeping
55	thioredoxin nuclear gene encoding mitochondrial protein	*Txn2*	NM_019913	housekeeping
56	hypoxanthine guanine phosphoribosyl transferase	*Hprt*	NM_013556	housekeeping
57	succinate dehydrogenase complex, subunit A, flavoprotein	*Sdha*	NM_023281	housekeeping
58	polymerase (RNA) II (DNA directed) polypeptide A	*Polr2a*	NM_009089	housekeeping

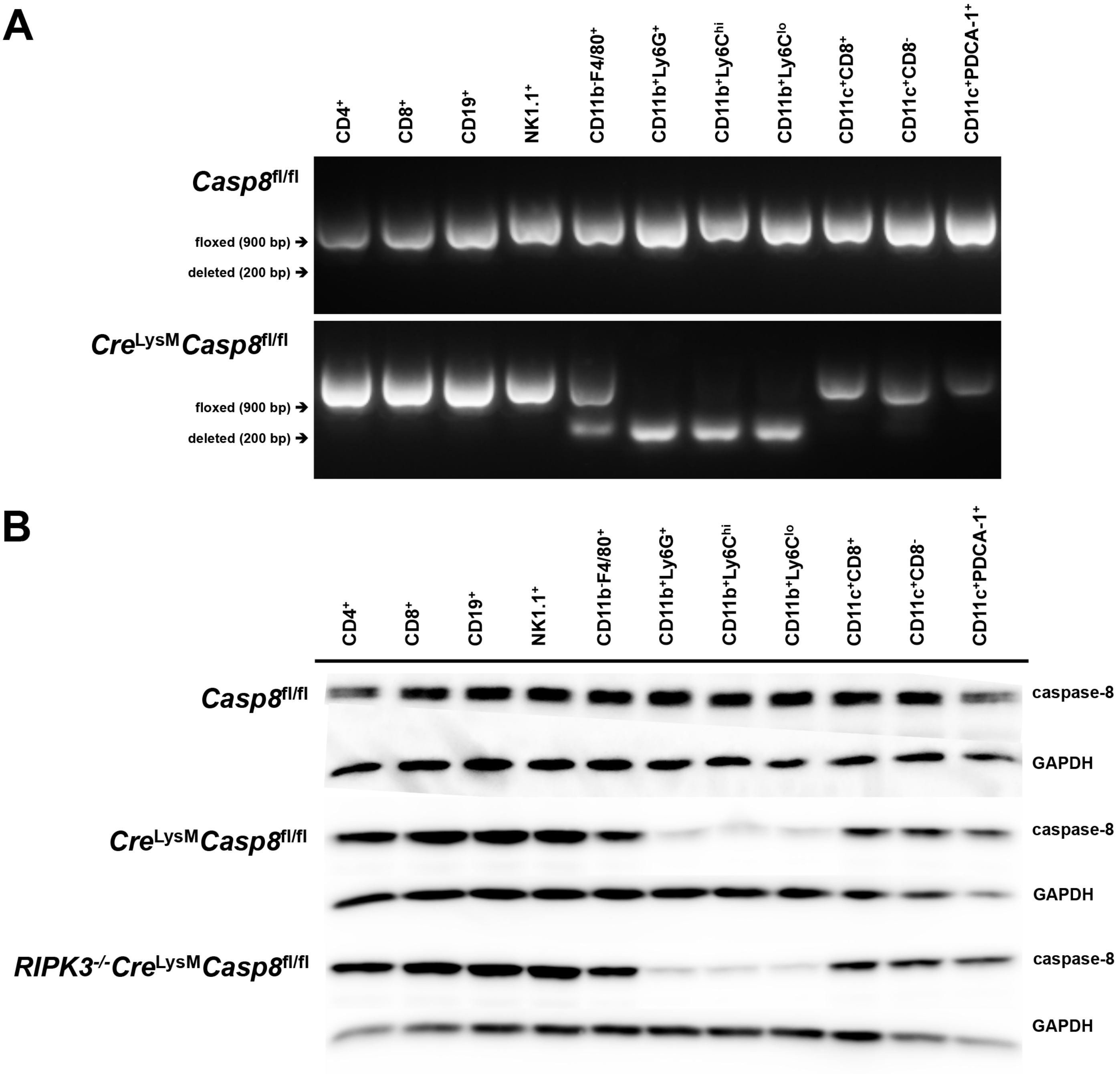

Figure 1. Genotype validation of splenocyte populations. (**A**) Splenocyte populations from *Cre*^{LysM}*Casp8*$^{fl/fl}$ mice were sorted as: CD4^{+} and CD8^{+} T-cells, B-cells (CD19^{+}), NK cells (NK1.1^{+}), red pulp macrophages (CD11b^{-}F4/80^{+}), neutrophils (CD11b^{+}Ly6G^{+}), monocytes/macrophages (CD11b^{+}CD11c$^{low/negative}$SSC^{low}F4/80^{+}) further subdivided into Ly6C^{low} and Ly6C^{high}, plasmacytoid DC (mPDCA-1^{+}B220^{+}CD11c^{intermediate}) and conventional DC (B220^{-}CD11c^{+}CD8^{+} and B220^{-}CD11c^{+}CD8^{-}), and subjected to PCR for *Casp8*^{floxed} and *Casp8*^{deleted} alleles. (**B**) Splenocyte populations sorted as above from *Casp8*$^{fl/fl}$*, Cre*^{LysM}*Casp8*$^{fl/fl}$ and *RIPK3*$^{-/-}$ *Cre*^{LysM}*Casp8*$^{fl/fl}$ mice were subjected to western blot analysis for caspase-8 and GAPDH protein expression.

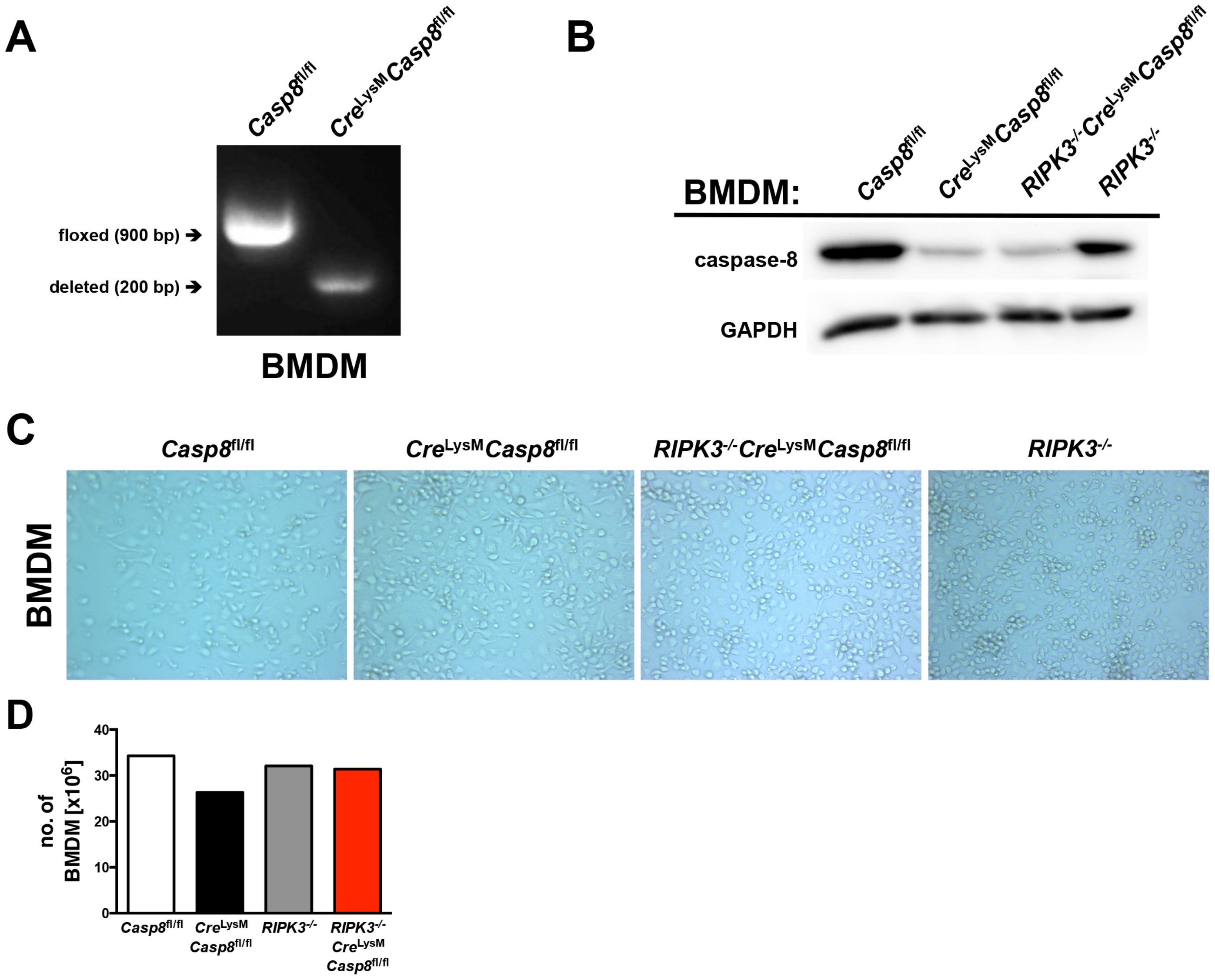

Figure 2. Genotype validation of BMDMs. (**A**) M-CSF-generated BMDMs from *Casp8*$^{fl/fl}$ and *Cre*^{LysM}*Casp8*$^{fl/fl}$ mice were subjected to PCR for *Casp8*^{floxed} and *Casp8*^{deleted} alleles. (**B**) M-CSF-generated BMDMs from *Casp8*$^{fl/fl}$*, Cre*^{LysM}*Casp8*$^{fl/fl}$, *RIPK3*$^{-/-}$*Cre*^{LysM}*Casp8*$^{fl/fl}$ and *RIPK3*$^{-/-}$ mice were subjected to western blot analysis for caspase-8 and GAPDH protein expression. (**C**) Representative growth of M-CSF-generated BMDMs from *Casp8*$^{fl/fl}$*, Cre*^{LysM}*Casp8*$^{fl/fl}$, *RIPK3*$^{-/-}$*Cre*^{LysM}*Casp8*$^{fl/fl}$ and *RIPK3*$^{-/-}$ mice. (**D**) Quantitation of M-CSF-generated BMDMs per mouse per genotype.

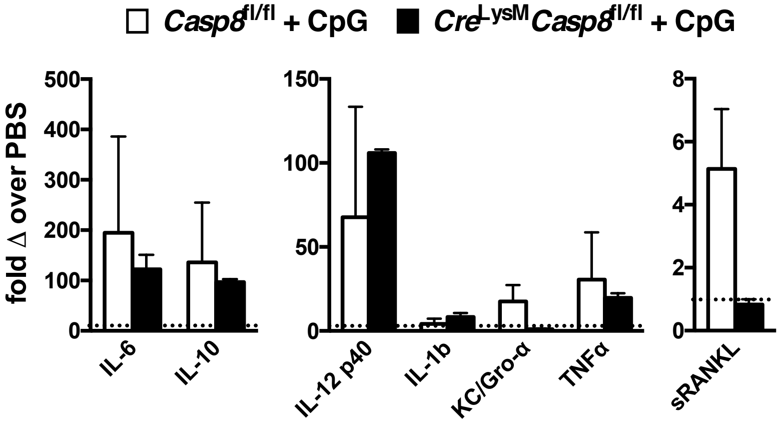

Figure 3. TLR9 *in vivo* activation induces upregulation of serum cytokines and chemokines at similar levels in *Casp8*$^{fl/fl}$ and *Cre*^{LysM}*Casp8*$^{fl/fl}$ mice. 3-month-old *Casp8*$^{fl/fl}$ (control) and *Cre*^{LysM}*Casp8*$^{fl/fl}$ mice (n=4) injected with CpG (200 μg/mouse) were evaluated 4 hours later for alterations in serum cytokine and chemokine levels.

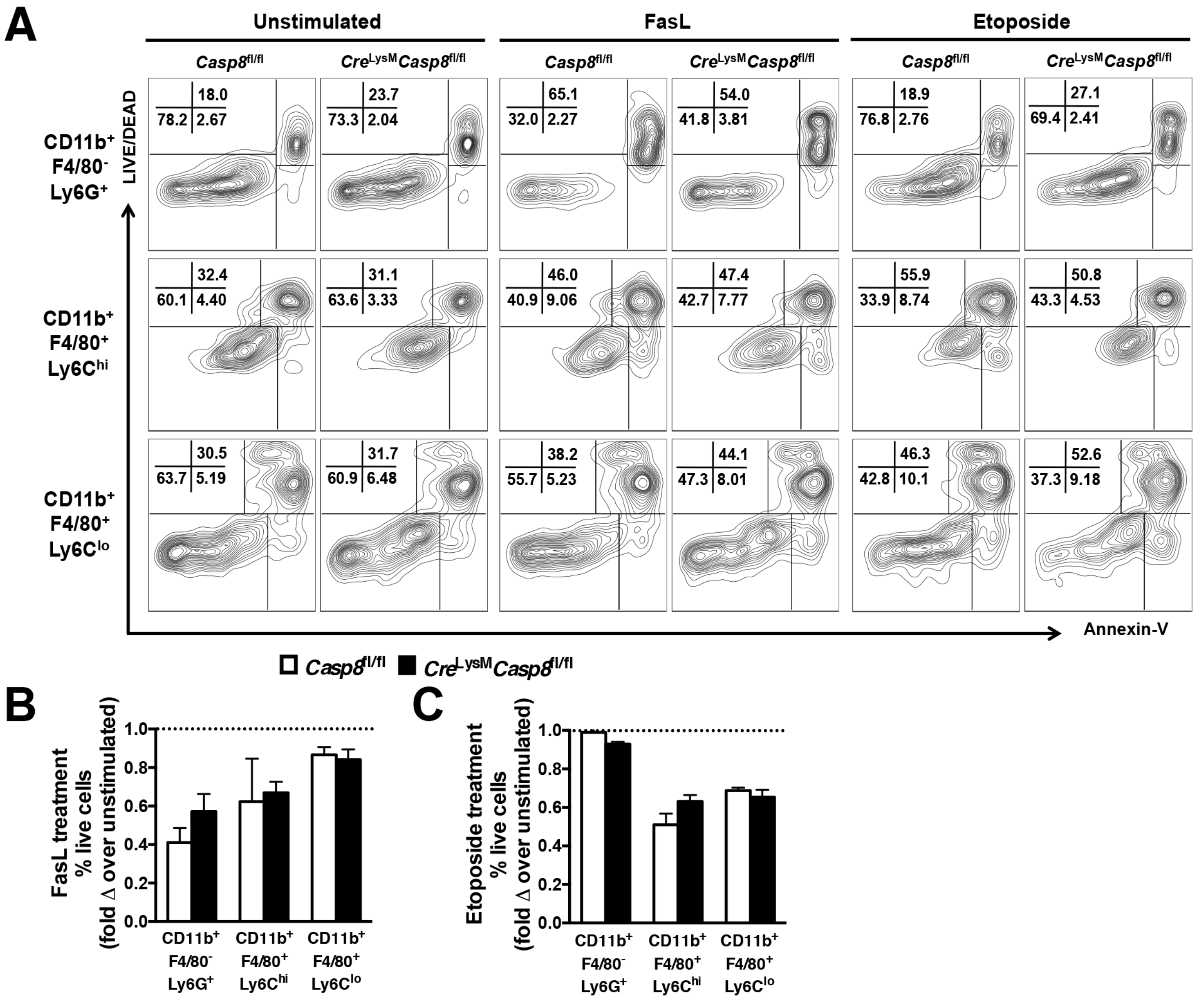

Figure 4. Caspase-8-deficient splenic myeloid populations are not predisposed to aberrant death. Total splenocytes from 2-3-month old *Casp8*$^{fl/fl}$ (control) and *Cre*^{LysM}*Casp8*$^{fl/fl}$ mice (n=3) treated with superFasL (100 ng/mL) or etoposide (10 μM) for 10 hours were stained with Annexin-V and Aqua live/dead. (**A**) Representative FACS plots and (**B-C**) quantitative graphs of the percent live myeloid populations treated with (**B**) superFasL or (**C**) etoposide relative to the unstimulated group. Data are represented as mean ± SD of biological triplicates and experiments were repeated twice. Data are compared by Mann Whitney test.

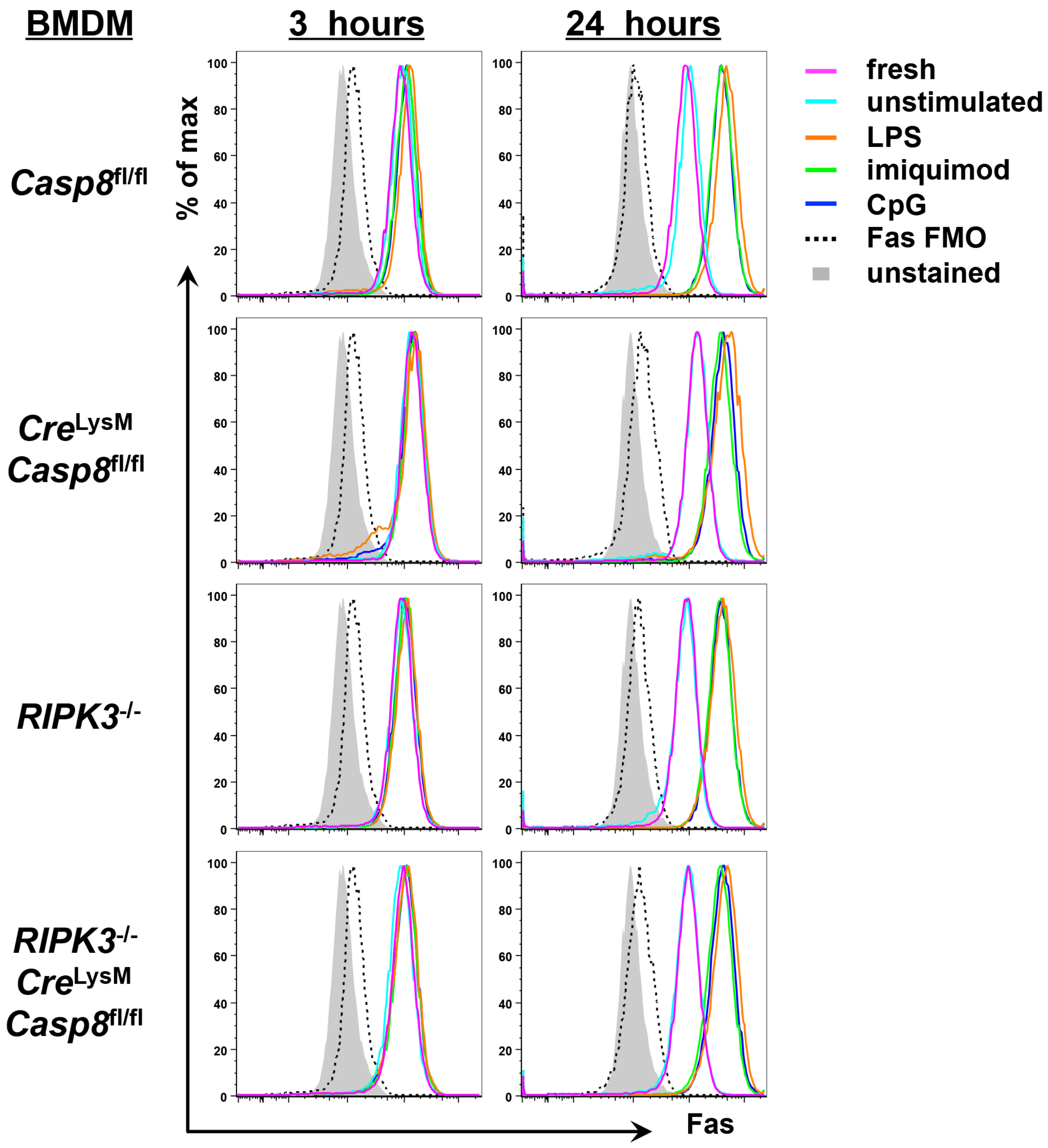

Figure 5. Caspase-8-deficient BMDMs express Fas. *Casp8*$^{fl/fl}$ (control), *Cre*^{LysM}*Casp8*$^{fl/fl}$, *RIPK3*$^{-/-}$ and *RIPK3*$^{-/-}$*Cre*^{LysM}*Casp8*$^{fl/fl}$ BMDMs treated with LPS (10 ng/mL), imiquimod (5 µg/mL) or CpG (5 µg/mL) for 3 and 24 hours were stained for Fas expression.

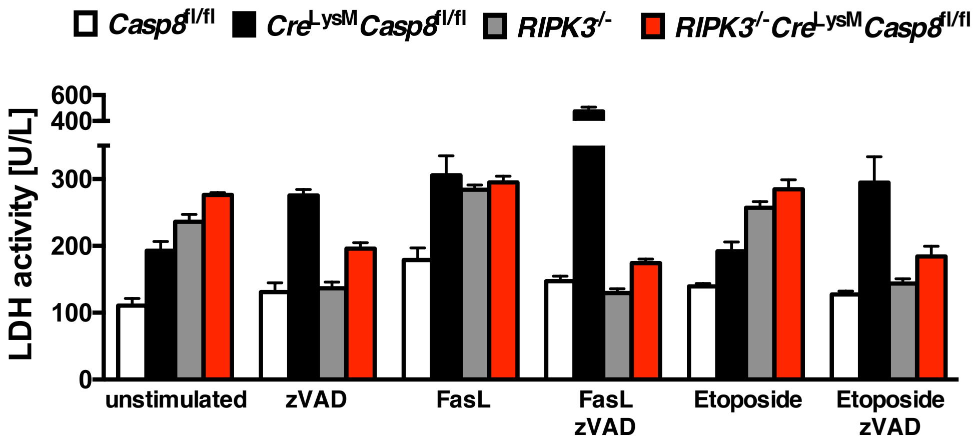

Figure 6. Caspase-8-deficient BMDMs undergo caspase-independent cell death in response to apoptotic stimuli. *Casp8*$^{fl/fl}$ (control), *Cre*^{LysM}*Casp8*$^{fl/fl}$, *RIPK3*$^{-/-}$ and *RIPK3*$^{-/-}$*Cre*^{LysM}*Casp8*$^{fl/fl}$ BMDMs were treated with superFasL (100 ng/mL) or etoposide (10 μM) ± zVAD-FMK (zVAD, 20 μM) for 48 hours. Supernatants were evaluated for LDH activity. Data are represented as mean ± SD of biological triplicates and experiments were repeated twice.

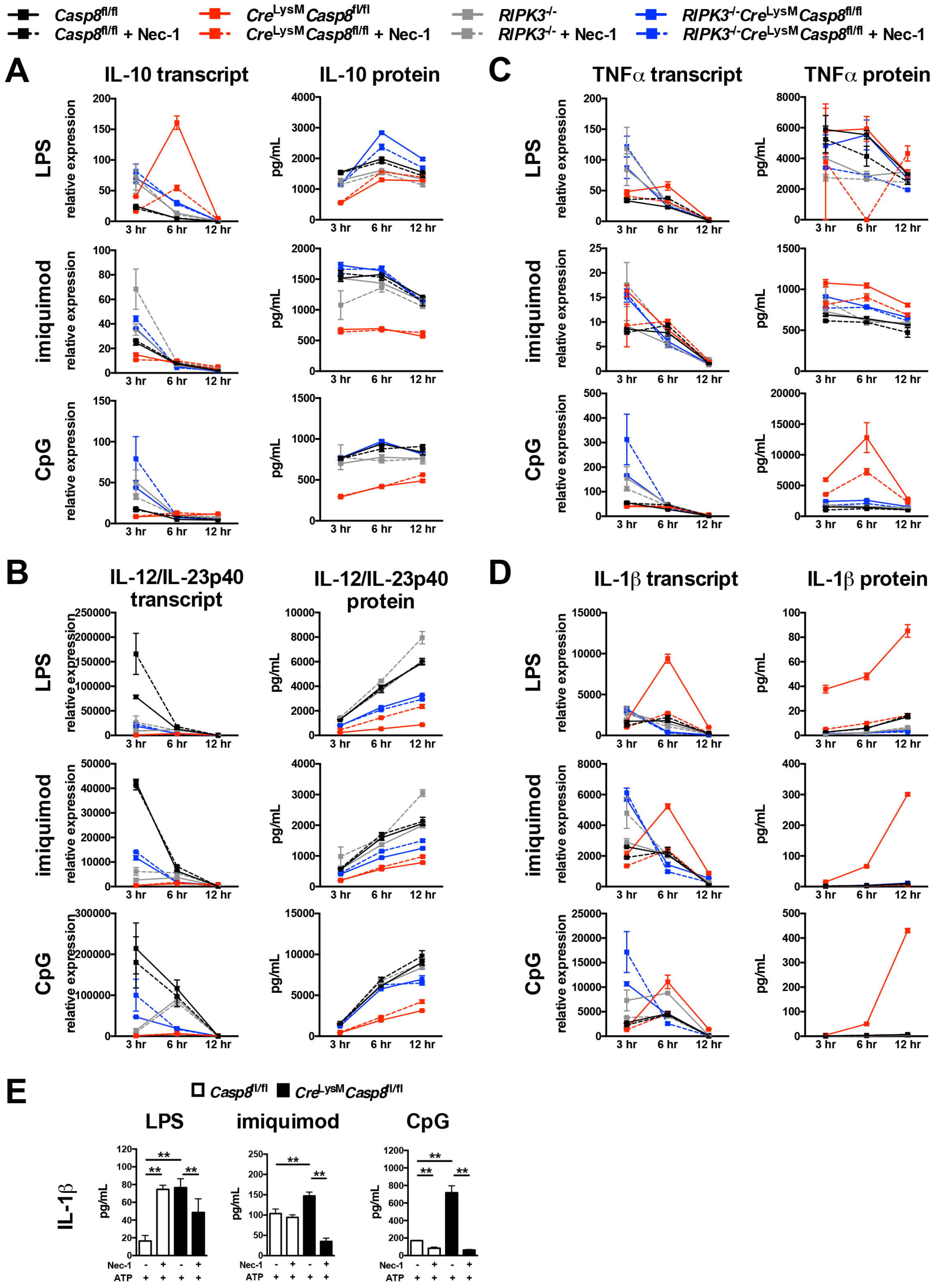

Figure 7. Caspase-8 deficiency in macrophages alters the response to TLR activation *in vitro*. (**A-D**) $Casp8^{fl/fl}$ (control), $Cre^{LysM}Casp8^{fl/fl}$, $RIPK3^{-/-}$ and $RIPK3^{-/-}Cre^{LysM}Casp8^{fl/fl}$ BMDMs were stimulated with LPS (10 ng/mL), imiquimod (5 μg/mL) and CpG (5 μg/mL) ± necrostatin-1 (Nec-1, 30 μM) for 3, 6 and 12 hours and evaluated for transcript and supernatant levels of (**A**) IL-10, (**B**) IL-12/IL-23p40, (**C**) TNFα and (**D**) IL-1β. (**E**) Control and $Cre^{LysM}Casp8^{fl/fl}$ BMDMs were stimulated with LPS (10 ng/mL), imiquimod (5 μg/mL) and CpG (5 μg/mL) ± necrostatin-1 (Nec-1, 30 μM) for 6 hours and ATP (5mM) was added for 45 minutes to evaluate IL-1β levels in supernatants. Data are represented as mean ± SD of biological triplicates and experiments were repeated twice. Data are compared by Mann Whitney test: **, $p<0.005$.

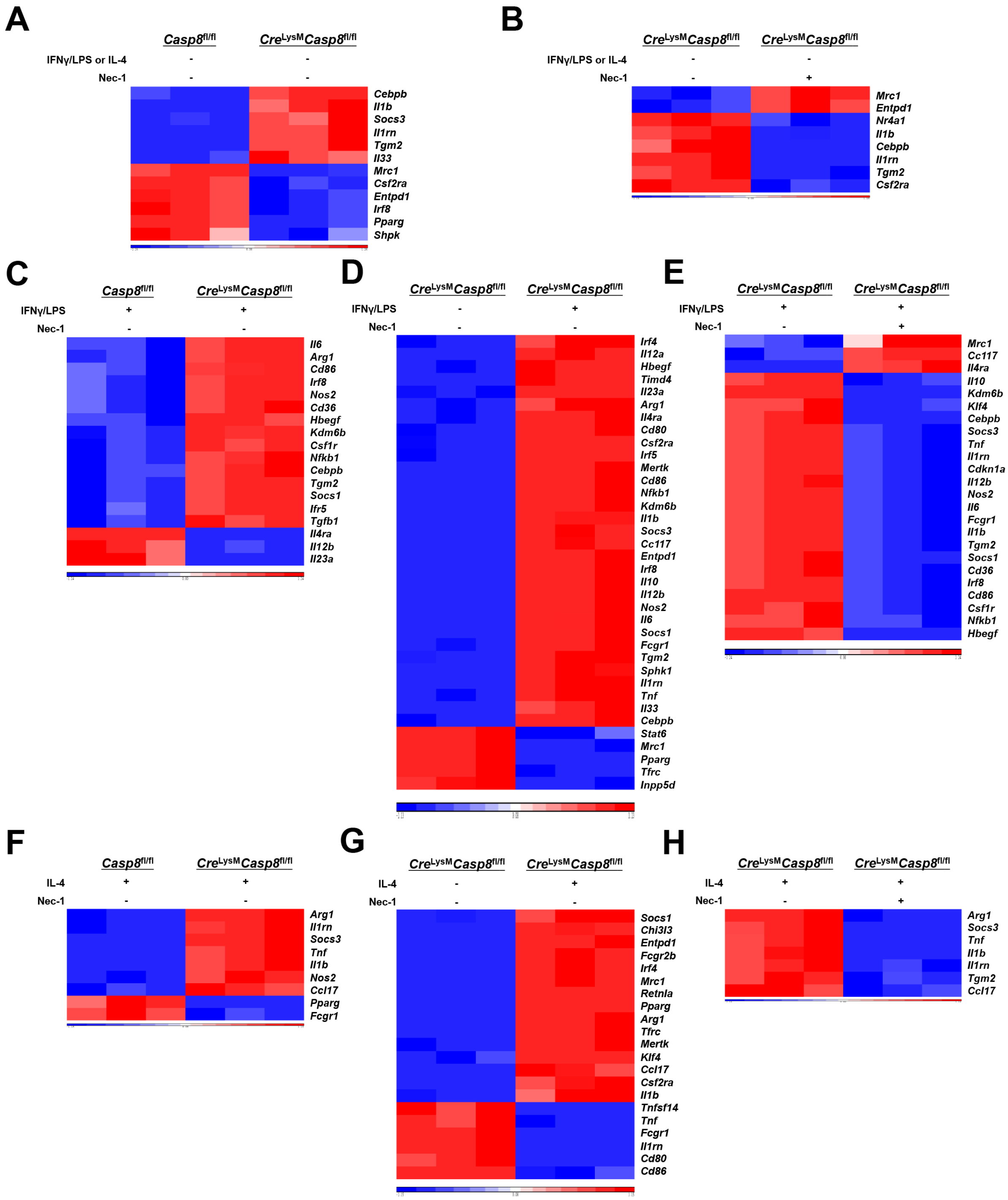

Figure 8. Caspase-8 deficiency in macrophages alters the genetic profile in response to macrophage polarization *in vitro*. (**A-H**) $Casp8^{fl/fl}$ (control) and $Cre^{LysM}Casp8^{fl/fl}$ BMDMs cultured with M1-polarizing conditions [primed overnight with IFNγ (100 ng/mL) and stimulated for 3 hours with LPS (10 ng/mL)] and M2-polarizing conditions [stimulated for 24 hours with IL-4 (40 ng/mL)] ± necrostatin-1 (Nec-1, 30 μM). Heat maps depicting differential gene expression analysis of the following: (**A**) Unstimulated control BMDMs vs. unstimulated $Cre^{LysM}Casp8^{fl/fl}$ BMDMs. (**B**) Unstimulated $Cre^{LysM}Casp8^{fl/fl}$ BMDMs vs. unstimulated $Cre^{LysM}Casp8^{fl/fl}$ BMDMs + Nec-1. (**C**) M1-polarized control BMDMs vs. M1-polarized $Cre^{LysM}Casp8^{fl/fl}$ BMDMs. (**D**) Unstimulated $Cre^{LysM}Casp8^{fl/fl}$ BMDMs vs. M1-polarized $Cre^{LysM}Casp8^{fl/fl}$ BMDMs. (**E**) M1-polarized $Cre^{LysM}Casp8^{fl/fl}$ BMDMs vs. M1-polarized $Cre^{LysM}Casp8^{fl/fl}$ BMDMs + Nec-1. (**F**) M2-polarized control BMDMs vs. M2-polarized $Cre^{LysM}Casp8^{fl/fl}$ BMDMs. (**G**) Unstimulated $Cre^{LysM}Casp8^{fl/fl}$ BMDMs vs. M2-polarized $Cre^{LysM}Casp8^{fl/fl}$ BMDMs. (**H**) M2-polarized $Cre^{LysM}Casp8^{fl/fl}$ BMDMs vs. M2-polarized $Cre^{LysM}Casp8^{fl/fl}$ BMDMs + Nec-1.
